# Graphene-Based Nanomaterials for Dental Applications: Principles, Current Advances, and Future Outlook

**DOI:** 10.3389/fbioe.2022.804201

**Published:** 2022-03-10

**Authors:** Xiaojing Li, Xin Liang, Yanhui Wang, Dashan Wang, Minhua Teng, Hao Xu, Baodong Zhao, Lei Han

**Affiliations:** ^1^ Department of Oral Implantology, The Affiliated Hospital of Qingdao University, Qingdao, China; ^2^ College of Chemistry and Pharmaceutical Sciences, Qingdao Agricultural University, Qingdao, China

**Keywords:** graphene, graphene oxide, dentistry, dental implants, osseointegration, bone regeneration, dental prosthesis

## Abstract

With the development of nanotechnology, nanomaterials have been used in dental fields over the past years. Among them, graphene and its derivatives have attracted great attentions, owing to their excellent physicochemical property, morphology, biocompatibility, multi-differentiation activity, and antimicrobial activity. In our review, we summarized the recent progress about their applications on the dentistry. The synthesis methods, structures, and properties of graphene-based materials are discussed. Then, the dental applications of graphene-based materials are emphatically collected and described. Finally, the challenges and outlooks of graphene-based nanomaterials on the dental applications are discussed in this paper, aiming at inspiring more excellent studies.

## Introduction

Oral health is quite important because it can deeply affect human health and quality of life (Lamster, 2021). However, World Health Organization (WHO) had reported that over 70% world’s population suffered the dental-related diseases in 2016 (Gordon and Donoff, 2016). At the 74th World Health Assembly of WHO in 2021, oral health has been highly concerned (Lamster, 2021). The main common oral diseases include dental caries, periodontal diseases, tooth loss, and oral cancer. Nowadays, maintaining oral health is challenging. Although, many techniques and methods have been adopted to treat oral diseases, yet there was no ideal method. To improve these methods, many kinds of biomaterials have been applied.

Tissue defects (especially bone defect) caused by traumas, infections, or tumors are one of the most common diseases in dental field (Liu et al., 2020). Currently, many efforts have been taken to repair tissue defects. Moreover, the regeneration of dental-like tissues were harder than tissues like bone, and muscle, because cementum regeneration is slow and pulp regeneration is hard. Besides, the regeneration of alveolar bone is relative active and rapid (Riccardo et al., 2018). Tissue engineering is commonly considered as a superior treatment strategy, where scaffolds played a vital role. Nowadays, most commercial biomaterials lack osteoinductive properties, which are very important for bone regeneration (Wu et al., 2017). Therefore, it is vital and urgent to discover an osteoinductive biomaterial for bone reconstruction.

In the field of dentistry, dental implants have been widely applied to restore the missing teeth due to their various advantages. It is well known that osseointegration is the gold standard for successful dental implantation. Titanium and its alloy have been applied as dental implant materials because of its good biocompatibility, mechanical properties, and so on. Except for all its merits, titanium implants also have failures due to the poor osseointegration. Therefore, it is important to improve the performance of titanium dental implants, and modifications of dental implant surfaces played an important role (Steflik et al., 1999). Various biomaterials have been widely applied to enhance the osteogenic properties of dental implants. In addition, the peri-implantitis is also the main failure reason for dental implants (Hideaki et al., 2019). Therefore, it is of great importance to explore new excellent antibacterial surfaces of dental implant.

Nanomaterials have showed wonderful performances in improving the strength and resist wear of tooth fillers and sealants. Moreover, nanomaterials also performed excellent antimicrobial properties in the application of restorative materials (Sharan et al., 2017). Owing to the above advantages, outstanding nanomaterials are widely applied in the dental fields of restorative materials, adhesives, cements, primers, and so on.

**FIGURE 1:**
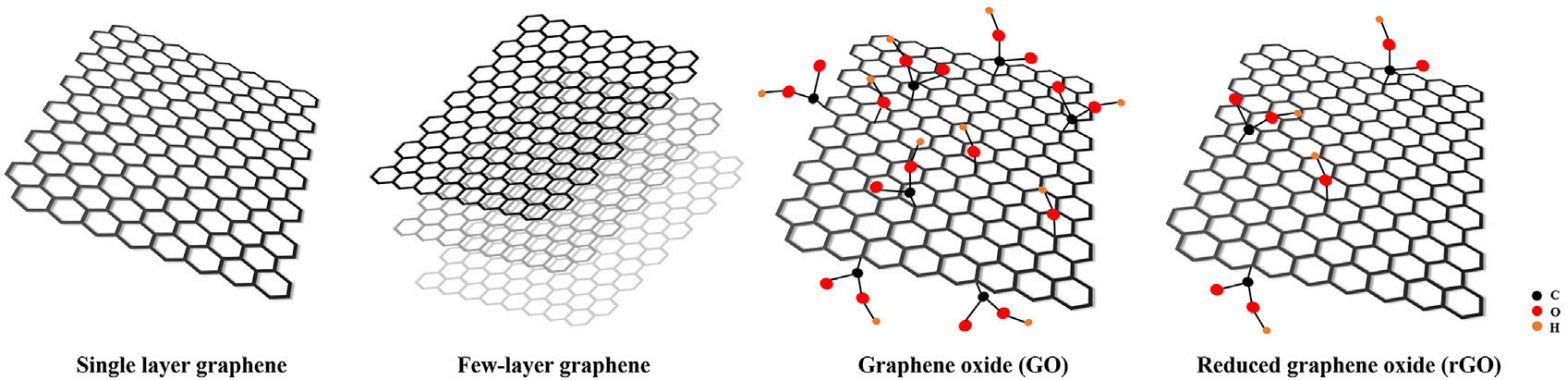
The structures of graphene-based materials.

Among various nanomaterials, graphene, as a promising two-dimensional (2D) carbon-based nanomaterial, is the thinnest and strongest material. In 2004, it was first isolated by Novoselov and Geim using mechanical exfoliation with a sticky tape and they won the Nobel Prize in 2010 (Novoselov et al., 2004). Graphene-based materials could be divided into four categories: single-layer graphene, few-layered graphene, graphene oxide (GO) and reduced graphene oxide (rGO) (Figure 1) (Bei et al., 2019). Owing to perfect physical properties, well electrical conductivity, and excellent biocompatibility, graphene and its derivative have attracted much attention in the field of medicine and biomedical fields. Moreover, graphene and its derivatives have also aroused great attentions in the field of dentistry and tissue engineering, dental implant coatings, bone cements, resin additives, and tooth whitening.

**FIGURE 2:**
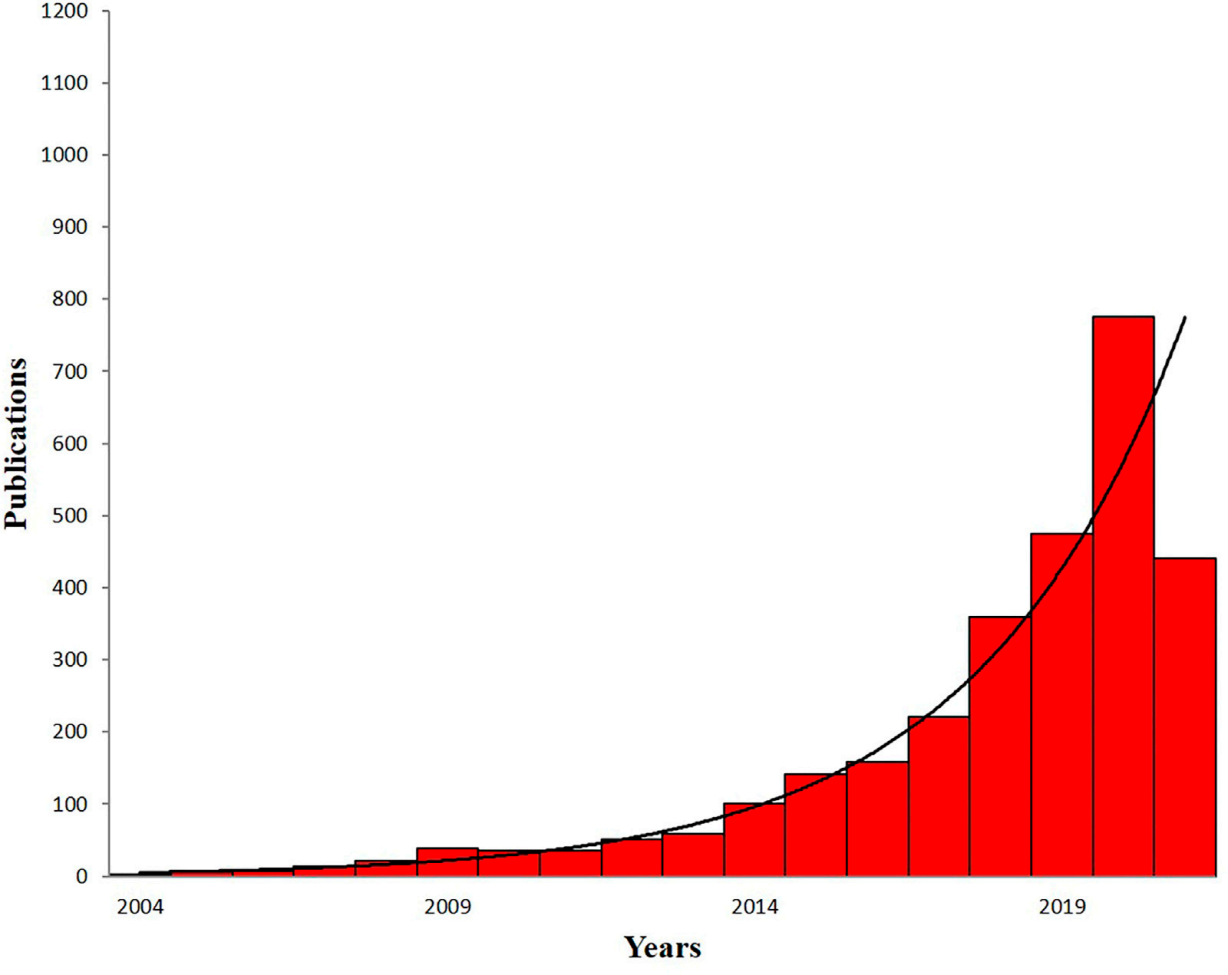
The numbers of published papers on the application of graphene-based materials in dental field by the end of June 2021. Data are from PubMed.

During the past 20 years, significant advances have been achieved in regulating properties of graphene and its derivatives, elucidating their underlying mechanisms, and broadening potential applications. Up to now, there are more than 2,942 studies related to graphene-based materials for dental applications. Although numerous excellent reviews have been published, most of those reviews were mainly focused on certain specific aspect. Therefore, a comprehensive review is needed to summarize and analyze all the progress, especially the achievements from more than 2,271 research papers published in the past 5 years (Figure 2). Such an analysis is necessary to help researchers to better understand graphene-based materials. To highlight the recent progress, various types, performance, and the applications of graphene-based materials such as graphene, GO, and rGO are summarized in this review. Finally, the challenges and future perspectives of graphene-based materials are also discussed. The purpose of this review aimed at summarizing the dental application of graphene-based materials and proposing the challenges and prospects.

## Synthesis and Structure of Graphene and its Derivative

Graphene, as a promising one-atom thick, 2D carbon-based nanomaterial, is currently the thinnest and strongest material. GO and rGO are the two main derivatives of graphene. Graphene and its derivatives have similar structures but different functional groups, which could lead to the differences in the physical and chemical properties. In this part, the synthesis and structure of graphene and its derivatives will be discussed.

### Synthesis and Structure of Graphene

In 2004, the Novoselov and Geim group firstly isolated graphene by mechanical exfoliation method with a sticky tape (Novoselov et al., 2004). Graphene made up of sp^2^ hybridized carbon atoms arranged in honeycombed lattice, and the structure of graphene is composed of six-membered rings stacked in parallel shape with no chemical groups on the surface of graphene (Zhang et al., 2019). Graphene aroused great interest, owing to its high mechanical strength, large surface area, good conductivity, and so on (Du et al., 2020).

Graphene from mechanical exfoliation has high purity and low defect, yet the yield is very low. To enhance the yield of graphene, many synthesis methods have been developed. There are two main synthesis approaches: top-down approach and bottom-up approach (Huang et al., 2012; Liao et al., 2018). On the one hand, the bottom-up approach includes the direct synthesis of graphene from the carbon materials, such as chemical vapor deposition (CVD) methods, graphitization of carbon-containing substrates by high temperature annealing, solid-phase deposition (Guo et al., 2009; Xiao et al., 2011). On the other hand, the top-down approach involves micromechanical cleavage, chemical exfoliation of GO followed by reduction treatment, and liquid-phase exfoliation.

#### Mechanical Exfoliation

In 2004, Geim group firstly obtained monolayer graphene by mechanical exfoliation method (Novoselov et al., 2004). The mechanical exfoliation of graphene is realized with a sticky tape from graphite crystals. Then, the tape was treated with specific solvents (such as acetone), and then, graphene was desorbed and harvested (Phaedon and Christos, 2012). Although the resulting graphene was highly pure without any chemical groups and defects, the yield was very low (Phaedon and Christos, 2012).

#### Liquid-Phase Exfoliation

Liquid-phase exfoliation is a good technique for a small scale of synthesis of graphene. First, graphite suspension is prepared in an organic solvent to weaken the van der Waals forces between the graphite layers. The graphite was then stripped into graphene sheets by ultrasound at a certain voltage. After further centrifugation, large quantities of single and multilayer graphene were obtained (Ghuge et al., 2016). The graphene is pure but small, and the number of layer is uncontrollable. Moreover, the use of surfactants and organic solvents leads to environmental pollution. The residual surfactants are difficult to remove in the process of exfoliation graphene. N,N-dimethyl-form amide (DMF), N-methyl-2-pyrrolidone (NMP), and dichlorobenzene (DCB) are the most frequently used organic solvents. However, they are toxic and detrimental to cells (Liao et al., 2018).

#### Chemical Vapor Deposition

CVD, one of the most successful approaches, has been widely employed for the low-cost and high-yield preparation of high-quality monolayer or few-layer graphene, where a large area monolayer graphene film formed on the metal (Ming et al., 2014). As for the synthesis process, methane, ethane, or propane is pyrolyzed at high temperature to form carbon onto the metal foils, such as Cu, Ni, Fe, Pt, and Ru. Then, the graphene film formed from free carbon atoms (Liao et al., 2018).

#### Chemical Exfoliation

Among various methods, chemical methods are one of the most productive ways in the synthesis of graphene-based materials. GO is firstly obtained by the Hummers method, which involves stirring or ultrasonic reaction of graphite with sulfuric acid, sodium nitrate, and potassium permanganate in water. Then, GO is kept at 1,000°C to exfoliate GO. GO is then reduced to rGO with reducing agents. Finally, rGO is converted to graphene by thermal or chemical treatments. However, it is difficult to remove all the oxygen-containing molecules in the GO. Besides, the long processing times and the toxic gases such as NO_2_ and N_2_O_4_ are also adverse factors during the synthesis progress.

#### Epitaxial Graphene

Epitaxial graphene could be fabricated on the SiC wafers under ultra-high vacuum and high temperatures. During the process, Si atoms on the surface of SiC wafers are sublimated and carbon domains are stayed on the SiC wafer surface, eventually forming graphene (Norimatsu and Kusunoki, 2014). However, because of the simultaneously growth of graphene in different position, the as-prepared graphene is not homogenous compared to other exfoliation methods.

In addition, Nickel diffusion approach is also a good alternative to SiC crystals. Nickel has a lattice structure similar to graphene with a thin nickel layer evaporated onto a SiC crystal. Being heated, the carbon diffuses through nickel layer and forms a graphene layer on the surface. The above methods make it easier to detach graphene layer from SiC crystal (Park and Ruoff, 2009).

GO exfoliation is also involved in other method such as laser ablation, anodic bonding, and photoexfoliation (Ansari et al., 2019). Laser ablation is the use of laser energy to peel graphene. In addition, the density of laser plays an important role in dominating the thickness and quality of graphene flakes (Kazemizadeh and Malekfar, 2017). Moreover, electrochemical exfoliation of graphite could obtain graphene nanosheets (Liu et al., 2008).

### Synthesis and Structure of GO

GO, achieved by the oxidation of graphene, presented various reactive oxygen functional groups (e.g., hydroxyl, carboxyl, and epoxy groups) located on the basal plane and the edges of GO, which are beneficial to combine different biomolecules such as proteins and drugs. The function of the above groups aims at interacting with proteins in the form of covalent, electrostatic, and hydrogen bonding. Considering the structure of GO, the amount of carbon atoms in GO has declined to 40%–60%, which are replaced with oxygen atoms. Therefore, GO displays the hydrophilic property. Although GO has shown more hydrophilic than graphene, GO displays defective structure, poor insulating property, and mechanical property (Reina et al., 2017).

The most well-known method of GO synthesis was proposed by Hummers and Offeman in 1958, in which graphite was oxidized by potassium permanganate and sulfuric acid (Hummers and Offeman, 1958). In the Hummers method, NaNO_3_ and KMnO_4_, as oxidants, were dissolved in concentrated H_2_SO_4_ to oxidize graphite. However, the Hummers method has the drawbacks of generation of toxic gas such as NO_2_ and N_2_O_4_, residual nitrate (Yu et al., 2016). To solve this problem, Kovtyukhova et al. used K_2_S_2_O_8_ and P_2_O_5_ to peroxide the graphite (Kovtyukhova et al., 1999). The mixture was then thermally isolated and cooled at room temperature for 6 h. Then, the mixture was rinsed with water until neutral pH. Finally, GO was obtained by the Hummers method. GO with high degree of oxidation was obtained by the oxidation of KMnO_4_ and NaNO_3_. In addition, Marcano et al. used KMnO_4_ rather than NaNO_3_ from the Hummers method. In addition, a mixture of H_2_SO_4_/H_3_PO_4_ was further used to enhance the effect of oxidization (Marcano et al., 2010). The modified method showed many advantages, such as high yields, no generation of toxic gas, and the controlled temperature. As we know, oxidization is the most important factor for fabricating the GO. Furthermore, Li et al. fabricated a high quality of GO with a green method within 1 h (Peng et al., 2015). The abovementioned improved method obtained a large-scale of graphene by using a strong oxidant K_2_FeO_4_ without producing heavy metals and toxic gases.

### Synthesis and Structure of rGO

rGO is mainly acquired by the reduction of GO. After reduction, the structures and properties of rGO have been greatly changed. Many oxygen-containing groups are removed from GO, and the sp^2^ structure of GO was restored to some extent, achieving a low carbon to oxygen ration (Lazauskas et al., 2018). As a result, the electrical conductivity is significantly enhanced. However, the conductivity of rGO is still inferior to graphene because of the residual oxygen and defects structure during the synthesis of GO.

The rGO could be obtained by the reduction of GO with different reduction conditions such as chemical reduction, thermal reduction, photochemical production, photothermal reduction, biological reduction, and electrochemical reduction (Cote et al., 2009; Guo et al., 2009; He et al., 2011; Mao et al., 2010; Williams et al., 2008; Yin et al., 2010). Among them, chemical reduction of GO is the most widely used technique to synthesize rGO with hydrazine, ascorbic acid, and bovine serum albumin (BSA) as reducing agents (Phaedon and Christos, 2012) (Rai et al., 2020) (Liu et al., 2010). Hydrazine is widely used in the chemical reduction of GO, but it is toxic and detrimental to cells. To overcome the limitations of these toxic-reducing agents, various green reduction agents have been investigated. For example, Cherian et al. used ascorbic acid as a reducing agent and stirred for 2 h in 95°C water bath to produce high-yielding black rGO (Cherian et al., 2019). Except for chemical reduction, thermal reduction of GO is employed under vacuum inert or other conditions suitable for reduction between 300°C and 2,000°C (Pei and Cheng, 2012; Wang et al., 2017). Photothermal reduction of GO can also be produced using laser with wavelengths under 390 nm (Lazauskas et al., 2018). Moreover, Li et al. reported that microwave heating could be used for the reduction of rGO in microwave ovens (Li et al., 2010).

## Dentistry-Related Property of Graphene and its Derivatives

### Biocompatibility and Cytotoxicity

To develop the application of graphene-based materials in dentistry, it is necessary to evaluate the biocompatibility and cytotoxicity of graphene-based materials (Olteanu et al., 2015). Many researchers have been discussed the cytotoxicity of graphene and its derivatives. Up to date, the affected factors involved concentrations, surface functionalization, and so on.

Many studies have shown a dose-dependent effect on the biocompatibility and toxicity of graphene and its derivatives. Some researchers showed that the toxicity of GO to fibroblast cells was little when the concentration of GO was lower than 20 μg/ml (Kan et al., 2011). Whereas, the cytotoxicity of GO increased when the concentration was up to 50 μg/ml. Wang et al. investigated the cytotoxicity of GO in mice and the results demonstrated a dose-dependent toxic behaviors *in vivo* (Wang et al., 2011). When the concentrations of GO were 0.1 and 0.2 mg, there was no toxicity was detected. With the increase concentration to 0.4 mg, chronic toxicity was observed in mice.

Some studies have also focused on the effect of surface functionalization on cytotoxicity. Diana et al. investigated the cytotoxicity of GO, nitrogen-doped graphene (N-Gr), and thermally reduced GO (TRGO) on human dental follicle stem cells and analyzed the involved specific mechanism (Olteanu et al., 2015). The result showed the lowest cytotoxicity of GO and the highest cytotoxicity of TRGO. However, Malgorzata et al. also compared the viability of leukocytes with GO, rGO, and rGO-PEG (Podolska et al., 2020). The results showed that there was no significant difference in the viability of leukocytes at the concentration of 50 μg/ml, indicating that the surface functionalization had no effect on the cell viability.

When the biomaterials were implanted to the tissue, the inflammation would be provoked by the protein interactions. During this process, many factors such surface charge, topography, and chemical compositions were involved and affected the resorption of protein. Besides, many molecules also played an important role in the process of inflammation such as betaines (D'Onofrio et al., 2019). Moreover, the tissue inflammation caused by graphene-based materials should be attracted on much attention. Eriberto et al. had reported that titanium nanoparticles released from dental implants could cause the chronic inflammation on the soft and bone tissue around the dental implants (Bressan et al., 2019).

Therefore, when using graphene-based nanomaterials as the coatings of dental implants and so on, we should also focused on its effect on the surrounding tissue inflammation. In the study of Rosa et al., they confirmed that friction during the dental implants emplaces when the loads is over 400 mN (Rosa et al., 2021). However, they also investigated that graphene nanocoatings did not activate the high expression of inflammatory markers such as TNF-α from macrophages. Of course, more and more studies should be done to prove this result.

### Stimulation of Cell Differentiation

Ideal biomaterials in the tissue engineering show the ability to induce the adherence, proliferation, and differentiation of cells. Many *in vitro* studies have shown that graphene and its derivatives showed the multi-differentiation ability such as osteogenic differentiation and regeneration of dental pulp.

Many studies have proved that graphene-based materials have the potential in promoting the osteogenic differentiation of various cells including MC3T3-E1, bone marrow mesenchymal stem cells (BMSCs), periodontal ligament stem cells (PDLCs), dental pulp stem cells (DPSCs), etc. (Lee, Shin, and Lee et al., 2015a; Norahan et al., 2019; Xie et al., 2016; Zhou et al., 2016). The osteogenic differentiation of graphene, GO, and rGO has all been tested in different forms and synthesis methods. DPSCs, PDLCs, dental follicle progenitor cells (DFPCs), and BMSCs are used to induce the osteogenic differentiation. For example, Han et al. prepared a monolayer graphene on copper foils by CVD method and studied the ability to induce the osteogenic differentiation of DPSCs (Xie et al., 2016). The results showed that osteogenic genes and proteins runt-related transcription factor 2 (RUNX2), osteocalcin (OCN), and collagen (COL) were upregulated on graphene, after the culture of 14 and 28 days. Erika et al. fabricated GO-coated COL sponge scaffolds and evaluated the ability of bone formation in tooth extraction socket (Nishida et al., 2016). After the GO scaffolds were implanted into the tooth extraction sockets of dogs, the radiological density and histological results showed that the GO promoted bone formation was fourfold compared with the control. To further investigate the osteogenic ability of rGO, Lee et al. constructed rGO coating on the HAp composites, which confirmed the osteogenic effect of rGO on human mesenchymal stem cells (hMSCs), the improved mineralization of calcium and phosphate and enhanced ALP activity (Lee et al., 2015b, Shin, and Jin et al., 2015).

To investigate the ability of graphene-based materials on the dental pulp regeneration, some researchers had focused on the neurogenic differentiation of graphene-based nanomaterials. For example, Seonwoo et al. prepared nanofibers (NFs) with electrospinning technique by incorporated with rGO and polycaprolactone (PCL) and investigated their enhanced neurogenesis of DPSCs (Seonwoo et al., 2018). The results showed that NFs with rGO showed high expression of Tuj-1 (the early marker of neurogenesis) and NeuN (the late marker of neurogenesis).

Scaffolds are an important part in the bone tissue engineering. Similarly, periodontal tissue engineering also required scaffolds to achieve an ideal therapy for periodontitis. Kawamoto et al. studied the GO modified COL sponge scaffold and evaluated the regeneration of periodontal tissue *in vivo* (Kohei et al., 2018). The rat cranial defect model was constructed, and the results showed that the new formed bone in the GO scaffolds was more than that of the control. To stimulate the therapy of periodontitis in human, the class Ⅱ furcation defects of dog were conducted. CT results showed that the radiopacity of GO scaffolds was obviously increased. In addition, the histological results showed that more new formed alveolar bone was found and the furcation defect was filled without severe root resorption. More interestingly, periodontal ligament-like and cementum-like tissues were also occurred, showing an ideal therapy effect.

### Antibacterial Property

As an excellent biomaterial in dentistry, low cytotoxicity and multi-differentiation ability are necessary. Except for these, antibacterial property cannot be ignored. The antibacterial effect of graphene-based materials was firstly discovered by Hu et al. (Hu et al., 2010). Then, more and more researchers had confirmed the antibacterial effect. For example, Gholibegloo et al. found that the bacterial survival rate of *S. mutans* treated with GO, GO-carnosine (GO-Car), and GO-Car/hydroxyapatite (HAp) can decrease by 67%, 86.4%, and 78.2%, respectively (Gholibegloo et al., 2018). Many composites had been fabricated to study its antibacterial property, and some researchers fabricated the graphene-based materials into glass ionomer cements (GICs), polymethyl methacrylate (PMMA), and dental adhesive (Bregnocchi et al., 2017; Lee J.-H. et al., 2018; Sun et al., 2018).

## Dental Applications of Graphene-Based Materials

**FIGURE 3:**
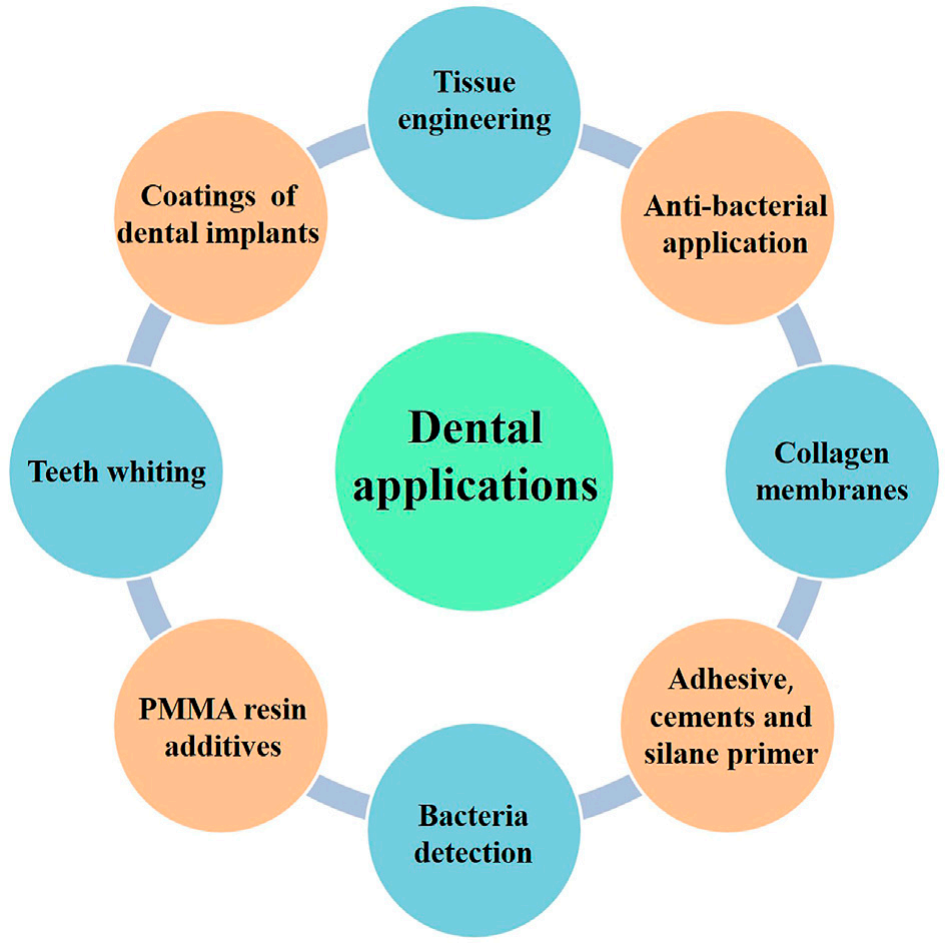
The multi-applications of graphene-based materials in the dental fields.

With the improved synthesis methods, expanded types of graphene-based materials, and engineered properties, various applications have been collected and discussed as follows (Figure 3). In addition, a main summary of graphene-based materials used in the dental field is given in Table 1.

**TABLE 1:** The graphene-based materials used in the main study of dental fields.

Applications	Types of graphene	Properties	Application types	References
Bone tissue engineering	Graphene/HA	Biomimetic mineralization	Scaffolds	Fan et al. (2014)
	Monolayer graphene	Osteogenic differentiation	Coatings	Xie et al. (2016)
	rGO/BCP	Osteogenic differentiation	Coatings	Jeong-Woo et al. (2017)
	rGO/HA	Mineralization	Scaffolds	Lee et al. (2015b)
	Silk fibroin/GO/BMP-2	Osteogenic differentiation	Scaffolds	Wu et al. (2019)
	GO/chitosan	Osteogenic differentiation	Scaffolds	Amiryaghoubi et al. (2020)
Dental pulp regeneration	Graphene dispersion	Neural differentiation	Scaffolds	Jelena et al. (2018)
	NFs/rGO/PCL	Neural differentiation	Scaffolds	Seonwoo et al. (2018)
	GO	Osteogenic differentiation	Scaffolds	Vinicius et al. (2016)
		Odontogenic differentiation		
Periodontal tissue regeneration	3D collagen sponge/GO	Osteogenic differentiation periodontal ligament-like tissue regeneration	Scaffolds	Kohei et al. (2018)
		Cementum-like tissue regeneration		
	Silk-fibroin/GO	Osteogenic differentiation	Scaffolds	Vera-Sánchez et al. (2016)
		Cementoblast differentiation		
Dental implant and abutment	Single-layer graphene sheets	Osteogenic differentiation	Coatings	Ming et al. (2018)
	GO/CS/HA	Osteogenic differentiation	Coatings	Suo et al. (2018)
	GO	Osteogenic differentiation	Coatings	Zhou et al. (2016)
	rGO/Dex	Osteogenic differentiation	Coatings	Jung et al. (2015)
	rGO nanosheets	Osteogenic differentiation	Coatings	Lu et al. (2020)
	GO/Minocycline hydrochloride (MH)	Antibacterial property	Coatings	Qian et al. (2018)
	GO/Ag	Antibacterial property	Coatings	Jin et al. (2017)
Collagene mmenber	GO	Roughness and stiffness	Coatings	Marco et al. (2017)
		Osteogenic differentiation		
		Inflammation effect		

### Tissue Engineering

Tissue engineering is widely used in the repair and regeneration of various defects caused by tumors, traumas, infections, and so on. It is well known that the scaffolds provide a platform for the attachment, proliferation, and differentiation of different stem cells in the tissue engineering. Many researchers proved that graphene-based materials were suitable for fabricating or coating for scaffolds in the tissue engineering.

#### Bone Tissue Engineering

Many studies confirmed that graphene-based materials can promote the osteogenic differentiation of various cells including osteogenic cells such as MC3T3-E1 and stem cells such as BMSCs, PDLCs, and DPSCs. As for bone tissue engineering, graphene-based materials could be used in various forms (such as composite powers, scaffolds, and surface coatings) (Yong et al., 2018).

Many studies have reported that graphene could stimulate different types of stem cells to form osteoblasts. Fan et al. reported that graphene/HAp composite sheet displayed good biomimetic mineralization (Fan et al., 2014). The similar osteogenic differentiation has been proved by Xie et al. (Xie et al., 2016). They prepared monolayer graphene with CVD and further evaluated the degree of mineralization and expression of osteogenic genes and proteins. They found that graphene induced RUNX2 and OCN expression and stimulated high expression of OPN and OCN in DPSCs. Moreover, Li et al. showed that graphene could induce the higher expression of osteogenic-related genes (OCN, OPN, BMP-2, and Runx2) compared with the control (Li et al., 2015). In addition, the enhanced osteogenic effect had also been confirmed by the high OCN expression at the protein level.

**FIGURE 4:**
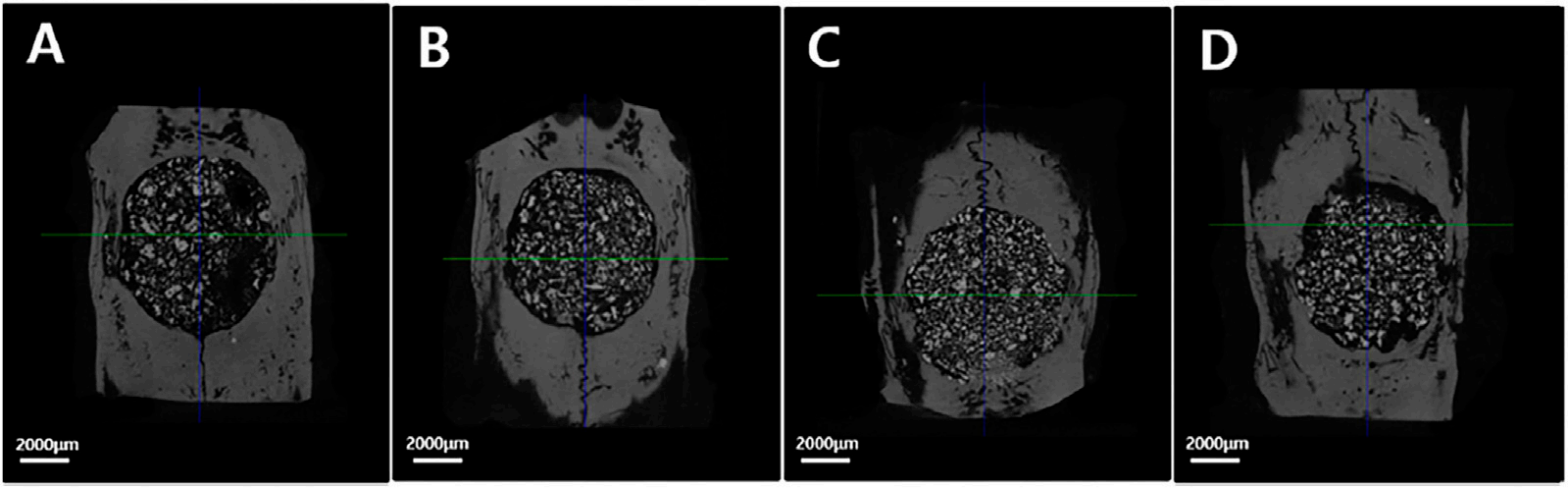
The micro-CT images of different groups. **(A)** Control group; **(B)** rGO2 group; **(C)** rGO4 group; **(D)** rGO6 group (Jeong-Woo et al., 2017). Open access, 2017, Jeong-Woo Kim.

**FIGURE 5:**
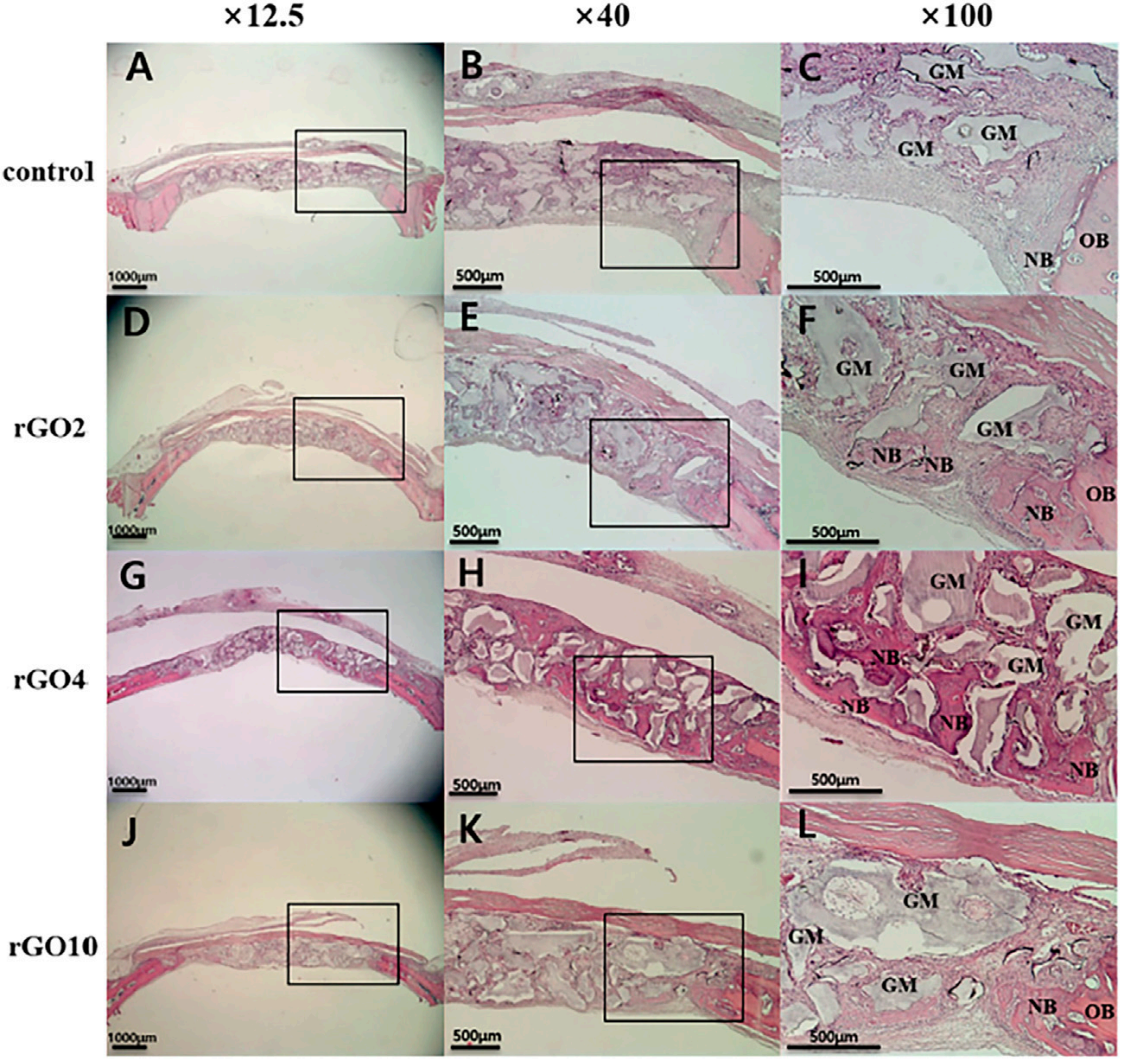
Histological images of specimens at 2 weeks after surgery. Black box is the interest area. **(A–C)** Control group; **(D–F)** rGO2 group; **(G–I)** rGO4 group; **(J–L)** rGO10 group. NB, new bone; OB, old bone; GM, bone graft material (Jeong-Woo et al., 2017). Open access, 2017, Jeong-Woo Kim.

**FIGURE 6:**
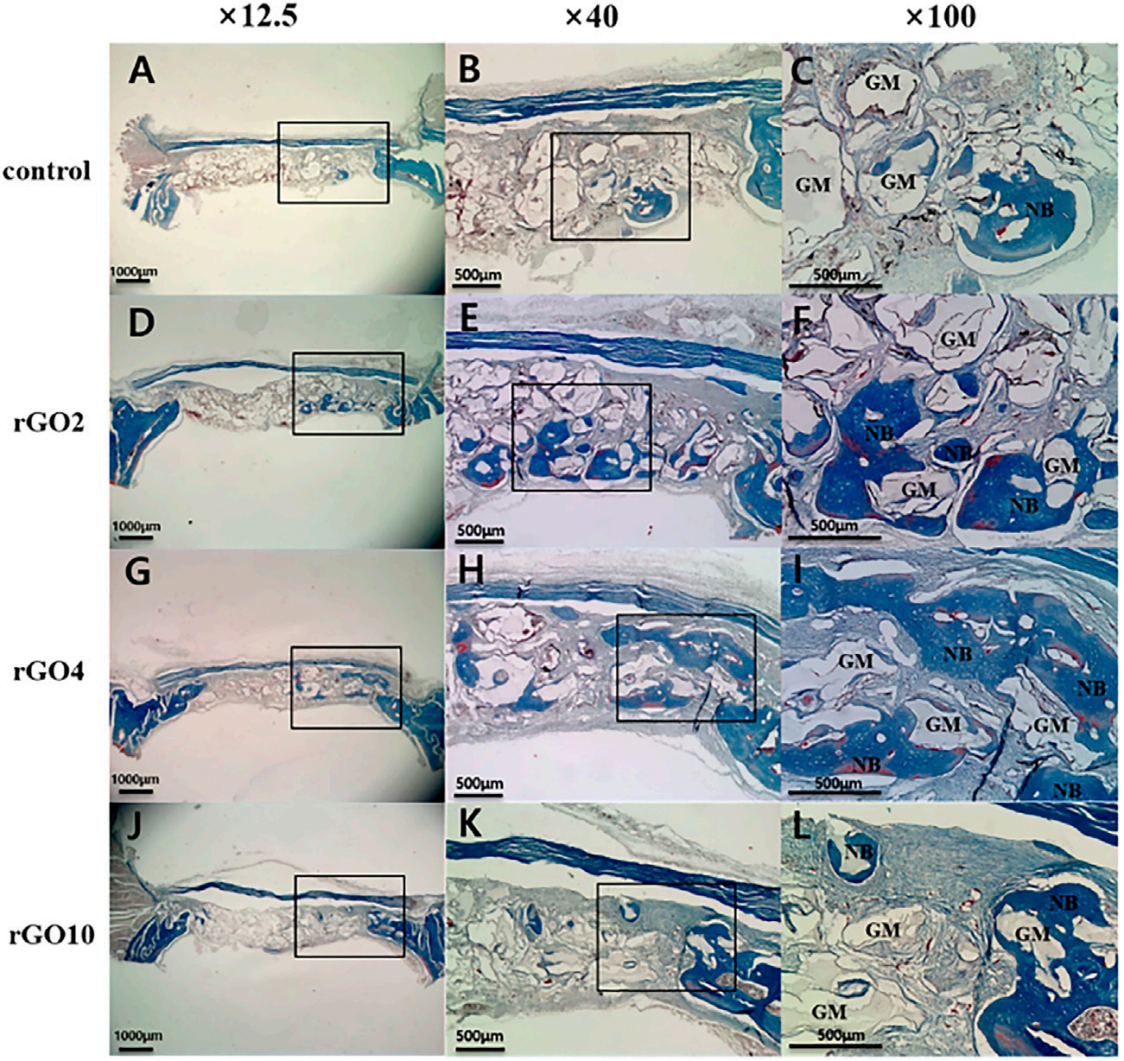
Histological images of specimen at 8 weeks after surgery with Masson’s trichrome staining. Black box is the interest area. **(A–C)** Control group; **(D–F)** rGO2 group; **(G–I)** rGO4 group; **(J–L)** rGO10 group. NB, new bone; OB, old bone; GM, bone graft material (Jeong-Woo et al., 2017). Open access, 2017, Jeong-Woo Kim.

To confirm the osteogenic differentiation of rGO, some researchers had fabricated rGO coatings and composites (Norahan et al., 2019). Kim et al. successfully fabricated rGO coating on the biphasic calcium phosphate (BCP) (Jeong-Woo et al., 2017). The results showed that the regeneration of new bone volume was higher in the rGO groups compared with the control (Figures 4–6). Except for coatings, composites are also the new strategy to confirm the osteogenic property of rGO. Lee and his colleges also constructed the rGO composites with HAps to further prove the promoted osteogenic properties of rGO (Lee et al., 2015a). The results showed that rGO/HAp composites at 10 μg/ml induced more calcium deposits at day 14 and 21, whereas the rGO group induced more calcium deposition at day 21. The rGO/HAp composite group also showed more mineralized bone nodules than the control at day 28 in von Kossa staining. Interestingly, Lim et al. creatively studied the synergistic effect of electromagnetic fields (EMFs) and reduced rGO on the osteogenic, neurogenic, and audiogenic differentiation of hMSCs (Lim et al., 2016). The ALP activity showed that rGO + PEMF group showed the highest ALP expression after incubation of 7 days.

To confirm the osteogenic differentiation of GO, diverse functionalizations of GO had been done such as scaffolds and nanosheets. As used for the scaffolds, Wu et al. modified the silk fibroin scaffold with GO-functionalized BMP-2 peptide. The results showed that the expression of OPN and COL-1 was upregulated by GO-P24 both in the osteogenic medium and non-osteogenic medium, demonstrating the synergistic effects of GO and BMP-2 (Wu et al., 2019). The experiment *in vivo* also confirmed the above results. Then, Azadian et al. also successfully fabricated the polyvinylidene fluoride (PVDF)–polyvinylalcohol (PVA)–GO scaffold and evaluated the ALP activity, calcium contents assays, and expression of osteogenic gene markers (Azadian et al., 2020). After 7 and 14 days, ALP expression and calcium content in PVDF-PVA group were higher than the other groups and PVDF-PVA-GO group, which also achieved the highest expression of Col-1, Runx2, and OCN in hiPSCs for 7 and 14 days. Besides, GO coatings are also constructed to prove the osteogenic property of GO. Recently, Amiryaghoubi and his co-workers reported a new injectable hydrogel composed with GO and chitosan (CS) as stem cells scaffolds for bone regeneration. According to the results, GO improved the mechanical properties of hydrogel and achieved an excellent performance in bone tissue engineering (Amiryaghoubi et al., 2020). Besides, Wei et al. testified the effect of pristine GO nanosheets on the proliferation and osteogenic ability of BMSCs by two biomimetic cell culture methods (Wei et al., 2017). When the concentration of GO nanosheets was 10 μg/ml, the proliferation of BMSCs with both seeding methods was inhibited at 3 days. Nowadays, phosphorene is a new 2D nanomaterials, which attracted great attentions after graphene (Tatullo et al., 2019a). The properties of phosphorene were quite similar to graphene-based materials, and they had wonderful biodegradability and biocompatibility compared with graphene-based materials. Liu et al. also investigated the synergistic effect of GO and phosphorene on the osteogenic differentiation (Liu et al., 2019).

#### Dental Pulp Regeneration

The neural and odontogenic differentiation induced by graphene-based materials were also observed. Graphene dispersion can be applied on the neural differentiation of stem cells of apical papilla (SCPAs) (Jelena et al., 2018). A good neuron-like cell bodies with long process were found in the graphene dispersion group. The graphene dispersion group showed high expression of NF-M and β III-tubulin and showed strong β III-tubulin and NeuN immuno-reactivity 7 days after nerve induction, indicating that graphene enhanced the neural differentiation of SCPAs.

To intensify the neural differentiation of graphene-based materials, Seonwoo et al. prepared a NFs incorporated with rGO and PCL by electrospinning technique and investigated the enhanced neurogenesis of DPSCs (Seonwoo et al., 2018). The results showed that NFs with 0.1% and 1% rGO exhibited high expression of Tuj-1 and NeuN, whereas NFs with higher rGO concentration only achieved intense expression of NeuN on days 3 and 7. To prove the neural differentiation of GO, Rosa et al. investigated the effect of GO on the differentiation of DPSCs (Vinicius et al., 2016). Except for the high expression of Runx2 and OCN, GO also significantly upregulated DMP-1 and DSPP with the odontogenic differentiation of DPSCs.

### Periodontal Tissue Regeneration

**FIGURE 7:**
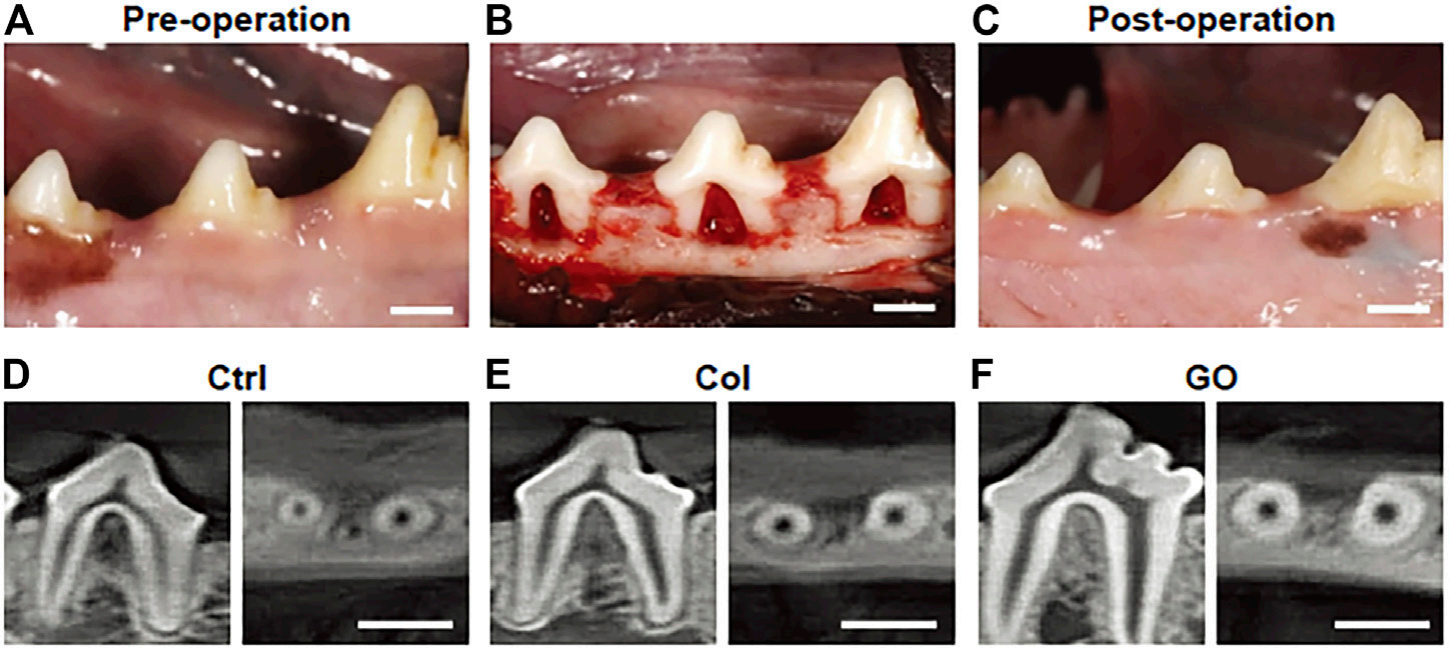
The photographs and CT images of class Ⅱ furcation defects of dog. **(A)** Pre-operation; **(B)** class Ⅱ furcation defects in the surgery; **(C)** postoperation; **(D–F)** CT images after 4 weeks surgery. **(D)** No implantation group; **(E)** collagen scaffold group; **(F)** GO scaffold group (Kohei et al., 2018). Free full-text article, 2018, Kohei Kawamoto.

**FIGURE 8:**
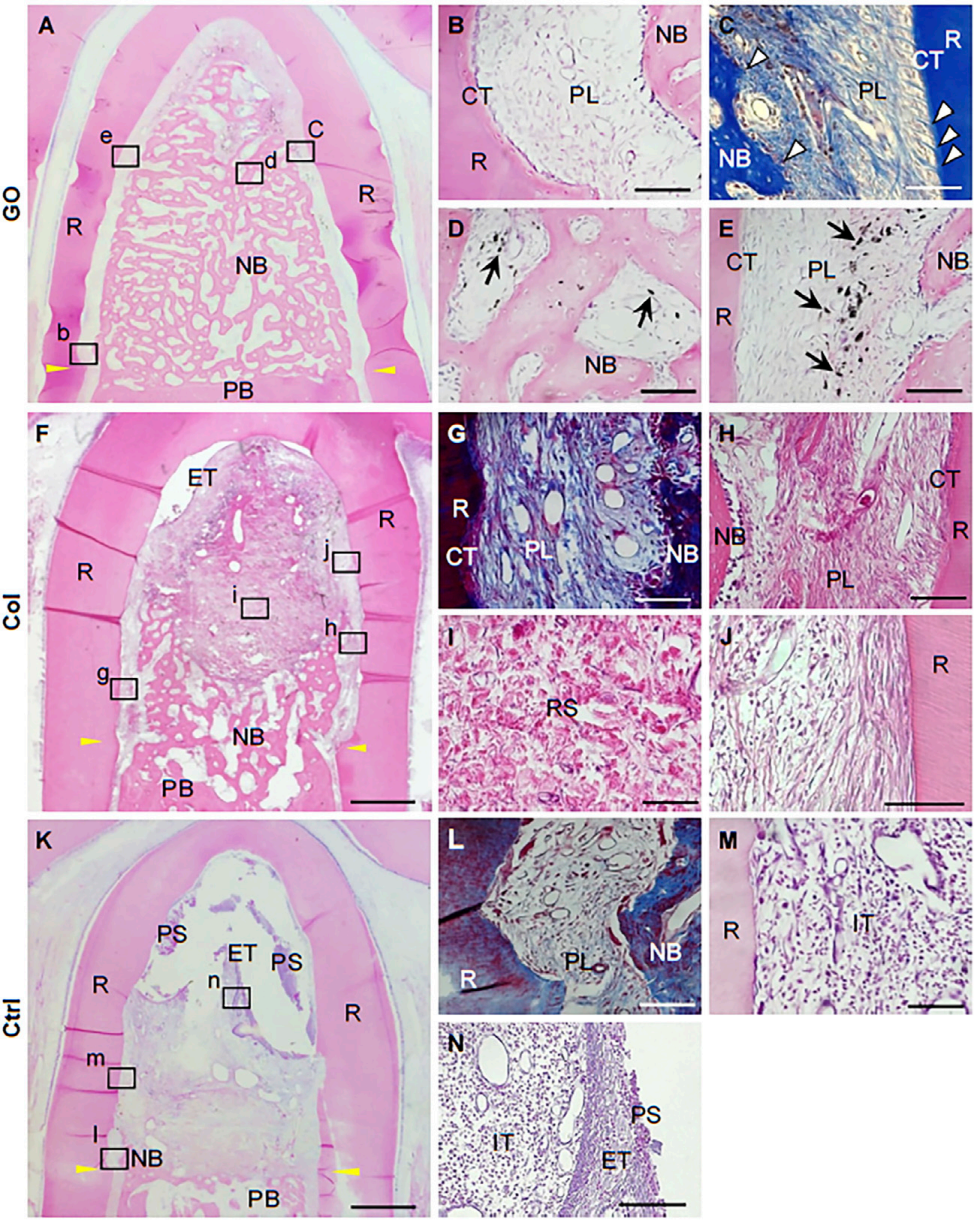
Histological images after GO scaffold implantation into class Ⅱ furcation defects of dog. **(A–E)** GO scaffold group; **(F–J)** collagen scaffolds group; **(K–N)** no implantation group. **(A)** Periodontal ligament–like tissue, cementum-like tissue, and alveolar bone with Sharpey’s fiber. **(B–E)** High magnification of the black box areas **(B–E)** in **(A)**. **(G–J)** High magnification of the black box areas **(G–J)** in **(F)**. **(K–N)** High magnification of the black box areas **(L–N)** in **(K)** (Kohei et al., 2018). Free full-text article, 2018, Kohei Kawamoto.

As we know, periodontitis is an inflammatory disease with dramatic destruction in periodontal tissue such as periodontal ligament, alveolar bone, and cementum. With the deterioration of periodontitis, the tooth faced the fate of losing, which led to many functional disorders. Therefore, it is quite urgent to regenerate and appealed many researchers. Compared with graphene and rGO, GO showed the hydrophilic surface and good dispersibility, which facilitated the absorption of some related proteins. Kawamoto et al. conducted 3D COL sponge scaffold with GO dispersion (Kohei et al., 2018). The histometric analysis showed that new formed bone in the GO group was, respectively, 2.7- and 2.3-fold greater than the control. In *in vivo* experiment, more new alveolar bone was found and filled the furcation defect. Even more interestingly, periodontal ligament-like, cementum-like tissue was also observed in the GO group (Figures 7, 8). To investigate the related mechanisms, Vera-Sánchez et al. had fabricated the GO and silk-fibroin composites and evaluated their osteogenic differentiation and cementoblast differentiation (Vera-Sánchez et al., 2016). The results of RT-PCR showed that the cementum related genes PTPLA/CAP and CEMP1 were highly expressed after incubating for 10 days. They further ascertained the cementoblast differentiation by evaluating the expression of cementum-related protein CEMP1 on days 10.

### Adhesives, Cements and Silane Primer

Adhesives and cements are two kinds of common materials in the dental restorations. Although they showed the advantages of aesthetic effect and high hardness, the problems of high polymerization shrinkage and bad antibacterial property limited their development. Silane primer played an important role in the bonding of zirconia.

Owing to various advantages of graphene-based materials, it has been applied to reinforce the properties of adhesive materials (Farooq et al., 2021). Graphene nanoplatelets (GNPs) are usually prepared as fillers of polymer dental adhesives because of the antimicrobial and antibiofilm activity. The nanocomposites filled with GNPs have been shown to effectively inhibit the active of *S. mutans* cells without compromising the bonding properties (Bregnocchi et al., 2017). Therefore, GNPs may be an ideal filler for dental adhesives, whose antibiofilm activity did not reduce mechanical performances.

When graphene nanosheets were added to two kinds of calcium silicate cements in powder form of different proportions, GNP–cement composites do well in shortening the bonding time and increasing the hardness of both cements. However, the bonding properties of one cement named Endocem Zr (ECZ) were impaired significantly, indicating that the addition of GNPs may improve the physico-mechanical properties of materials but not ideal for all materials in terms of bonding properties (Nileshkumar et al., 2017). Unlike gray GNPs, bright white fluorinated graphene (FG) may be a better filler in dentistry. FG has been used to the modification of GICs, presenting great advantages on the mechanical, tribological, and antibacterial properties. Compared with traditional GICs, the composites not only increase the Vickers micro hardness and compression strength but also decrease the friction coefficient. In the antibacterial properties, the GIC/FG composites achieve good antibacterial properties against *Staphylococci aureus* and *Streptococcus mutans* (Sun et al., 2018)*.*

Considering the bonding properties of resin composites to ZrO_2_, silane primers formed adhesive layer show the poorest mechanical properties (Fallahzadeh et al., 2017). Therefore, the corporation of GO sheets into the silane primers may be a good choice to improve the mechanical properties of the adhesive layer. The results showed that the addition of GO sheets significantly increased the shear bond strength between resin composite and ZrO_2_, improved the surface roughness, and slightly increased the water contact angle (Khan et al., 2019). Therefore, graphene-base materials are ideal filler for adhesive, cements, and silane primers with proper materials.

### Polymethyl Methacrylate Resin

PMMA resin has been used in prosthetic dentistry, especially complete and removable partial dentures over the past 80 years (Bacali et al., 2019), possessing many advantages such as easy manufacturing process, low cost, low modulus of elasticity, easy repair, and good aesthetics. However, the limitations of PMMA such as low mechanical properties, large polymerization shrinkage, and the poor inhibition of biofilms formation still exist (Bahra et al., 2013; Gad et al., 2019; Matsuo et al., 2015; Ruse and Sadoun, 2014). Recently, the graphene family has exhibited good mechanical and desirable antibacterial properties in different forms in other fields. Because of the mechanical effect of graphene on PMMA, Azevedo et al. has achieved the definitive maxillary full-arch rehabilitation by incorporating GO into the PMMA resin (Azevedo et al., 2019). After 8 months later, there were no mechanical, aesthetic, and other complications found, indicating that the addition of GO to PMMA resin would be a good choice for prosthetic rehabilitation. Bacali et al. reported PMMA with graphene-silver nanoparticles (Gr-Ag), and the mechanical properties, hydrophilic abilities, and the morphology of the composites were further evaluated (Bacali et al., 2019). The results showed that the compression parameters, bending, and tensile strength of the Gr-Ag fillers were significantly higher than the pure PMMA group, indicating that the addition of Gr-Ag improved the mechanical properties of PMMA resin. Moreover, Bacali and his co-workers also assessed the antibacterial properties of Gr-Ag–modified PMMA (Bacali et al., 2020), and the results confirmed that Gr-Ag–modified groups showed higher inhibition effect in all Gram-negative strain, *Staphylococcus aureus*, *E. coli*, and *Streptococcus mutans* (Bacali et al., 2020). In conclusion, graphene-based materials may be an ideal filler to promote the physical-mechanical and antibacterial properties of PMMA.

Meanwhile, graphene oxide nanosheets (nGO) have been used to improve the antimicrobial-adhesive effects of PMMA resin by Lee and his co-workers (Lee SM. et al., 2018). The immediate antimicrobial-adhesive effects showed that nGO-incorporated groups exhibited stronger antimicrobial effect against *C. albicans*, *E. coli*, *S. aureus*, and *S. mutans* after 1 h of attachment than only PMMA group except for 0.5% nGO-incorporated group against *E. coli* and *S. mutans*. Furthermore, PMMA with 2% nGO showed greater anti-adhesion effects than the PMMA resin after culturing for 28 days of *C. albicans*, indicating that the hydrophilicity of PMMA could be improved by the incorporation of nGO. Bacali et al. evaluated the antibacterial activity, cytotoxicity, monomer release, and mechanical properties of PMMA resin by adding graphene-Ag nanoparticles (G-AgNp) (Bacali et al., 2020). The results showed that G-AgNp–containing group displayed good antibacterial effects on both Gram-positive and Gram-negative strain and exhibited better antibacterial effect on Gram-positive strain *Staphylococcus* aureus than PMMA. Besides, Gram-negative strain *E. coli* also was sensitive to G-AgNp. Therefore, graphene family proved to be promising filler mixed with PMMA in antibacterial applications.

### Coatings for Dental Implants and Abutments

Titanium and its alloys have been widely used in dental implants, owing to their various advantages such as good biocompatibility, high mechanical property, and corrosion resistance (Fe Ridoun et al., 2017; Jeong et al., 2017; Xie et al., 2014). However, implant failure still occurs. Because of the poor osseointegration and peri-implantitis of titanium and its alloys (Berglundh et al., 2019; Smeets et al., 2014; Kordbacheh et al., 2019). Therefore, many surface modifications by graphene-based materials have been used to improve the bioactivities of titanium and its alloys (Barfeie et al., 2015; Chouirfa et al., 2018; Li et al., 2009; Souza et al., 2019).

It is well known that the osseointegration is the gold standard of the success of dental implants. Therefore, the new bone formation between dental implants and bone tissues is of great significance. In regard to the superior osteogenic differentiation of graphene in bone tissue engineering, they also attracted much attention in the modification of dental implant. Park et al. reviewed that the strategies of graphene-based modifications could be mainly divided into four categories: 1) a layer-by-layer assembly; 2) PMMA-mediated method; 3) electrophoretic deposition; and 4) APTES-induced method (Park et al., 2017).

To promote the osteogenic properties, many researchers have made many efforts. Gu et al. successfully constructed single-layer graphene sheets on the titanium substrates by PMMA-mediated method (Ming et al., 2018). The result showed that graphene sheets exhibited superior adhesion and proliferation properties of human gingival fibroblasts (hGFs), human adipose-derived stem cells (hASCs), and human BMMSCs (hBMMSCs) compared with the control. The osteogenic differentiation of hASCs and hBMMSCs was also improved by graphene sheets. Otherwise, the above study recommended that PMMA treated at 160°C for 2 h enhanced the adhesion strength between graphene and titanium. Meanwhile, Suo et al. successfully constructed a homogeneous GO/CS/HAp composites on Ti substrates by electrophoretic deposition, improving the osteogenic property of Ti substrates *in vitro* and osseointegration *in vivo* (Suo et al., 2018). Besides, some other researchers have tried the APTES inducement to fabricate the graphene coatings (Jung et al., 2015; Lu et al., 2020; Zhou et al., 2016). Zhou et al. modified the Ti substrates with 3-APTES and then immersed it in the GO solution for 24 h (Zhou et al., 2016). PDLCs were used to evaluate the cell attachment, morphology, proliferation, and osteogenic differentiation *in vitro*. All the results indicated that GO coating showed a positive effect on improving the bioactivity of PDLCs. Jung and his colleges prepared a dexamethasone (Dex)–loaded rGO coating on Ti13Nb13Zr (MPCR-TNZ) multipass rolled titanium alloy surface by using the spin coating technology after pretreatment with APTES, showing a stable long-term release behaviors of Dex (Jung et al., 2015). A possible mechanism is π-π stacking. In conclusion, the Dex-loaded rGO-MPCR-TNZ achieved an enhanced proliferation and facilitated differentiation of MC3T3-E1 cells into osteoblasts. Besides, an implantation of a prototype dental implant was modified with Dex-loaded rGO to an artificial bone block, demonstrating the feasibility of the stable surface modification for further clinical applications. Recently, Lu and his coworkers ascertained the improved osteogenic effect of rGO nanosheets on the surface of titanium (Lu et al., 2020). Compared with the control, rGO groups achieved more grow factors absorption especially BMP-2, IGF, and TGF-β. Moreover, rGO-modified group displayed a more stretch cell cytoskeleton and excellent osteogenic differentiation of BMSCs. In conclusion, graphene-based material is a good candidate for dental implant surface modification material, which can improve the osseointegration of implants with proper methods.

Moreover, the excellent antibacterial activity of graphene also captured a tremendous interest in dental implant surface coatings. It is generally accepted that the failures of dental implants are still the occurrence of bacterial infections. Therefore, the titanium surface antibacterial modification is very necessary. Qian et al. reported an electrostatically prepared GO coating that loaded the Minocycline hydrochloride (MH) on titanium (Qian et al., 2018). According to the results, GO-modified surface could prohibit the growth of *Staphylococcus aureus*, *Streptococcus mutans*, and *Escherichia coli*. Whereas MH-loaded GO coating showed superior antibacterial property with the synergistic effect of GO and MH, which were contact-killing and release-killing. Jin et al. prepared GO film and Ag nanoparticles on the titanium substrates by electroplating and ultraviolet reduction methods (Jin et al., 2017). In addition, GO-Ag-Ti composite exhibited a prominent antibacterial activity to *S. mutans* and *P. gingivalis* compared with the Ti substrate. Therefore, it can be inferred that graphene-based materials showed outstanding antibacterial activity when combined with proper materials.

Besides the titanium dental implants, zirconia has also been used for dental implant materials (Priyadarsini et al., 2017). To improve its inert properties, many surface modifications have been used. Graphene shows obvious merit in zirconia implant surface modifications (Cho and Ko, 2013; Schunemann et al., 2019). Li et al. reported a zirconia/graphene (ZrO_2_/GNs) composites, which were coated on the surface of zirconia by an atmosphere plasma spray technique (Li et al., 2014). The results indicated that ZrO_2_/GNs composites showed the excellent wear resistance and zirconia’s tribological behavior.

Peri-implantitis has become a common disease that threatened the survival rates of dental implants. Many strategies have been adopted to avoid the happening of peri-implantitis (Jansåker et al., 2010; Koldsland et al., 2018). Abutments are also an important part of implant system, and medicated abutments may be a good option for preventing the peri-implantitis. Qian et al. successfully fabricated a MH-loaded GO coating on the dental implant abutment and further evaluated the antibacterial activities and the adhesive ability of gingival fibroblasts with beagle dogs model (Qian et al., 2019). The results showed that MH/GO/Ti group achieved least bone loss, which could be negligible, whereas Ti and MH/Ti groups showed more bone loss by micro-CT analysis. Histological analysis showed that there were fewer neutrophils and more osteocytes in the MH/GO/Ti group than in the Ti and MH/Ti groups. Therefore, it can be inferred that MH-loaded GO films on abutment surfaces may be a superior option for prohibiting the progress of peri-implantitis in the future.

### Teeth Whitening

As we know, hydrogen peroxide (H_2_O_2_) has been widely utilized for in-office whitening for a long time. The H_2_O_2_ molecules can penetrate deep the teeth and carry out the bleaching process. However, the relative high concentrations of H_2_O_2_ caused some side effects such as tooth sensitivity and gingival irritation (Carey and Clifton, 2014; Kwon and Wertz, 2015). Therefore, many improved strategies have been made to accelerate the tooth whitening and decrease the side effects. Su et al. reported a cobalt (Co)/tetraphenylporphyrin (TPP)/rGO nanocomposite, which showed better tooth-whitening efficacy stained with dyes, tea, and betel nuts compared with the H_2_O_2_ only (I-Hsuan et al., 2016). In addition, H_2_O_2_ produces an extremely short lifetime of the active free radical. Therefore, to achieve a good bleaching effect, H_2_O_2_ must first penetrate into the teeth and quickly produce active free radicals. However, the Co/TPP/rGO nanocomposite can be used as a catalyst to produce more reactions between the staining molecules and H_2_O_2_, which accelerate the bleaching process. In summary, graphene-based materials are a promising catalyst for tooth whitening application with proper types and concentrations.

### Antibacterial Property

Bacterial biofilms formation plays an important role in the development of dental caries, periodontitis, and peri-implantitis (Berglundh et al., 2019; He et al., 2015; Kulshrestha et al., 2014; Slots, 2017; Ucja et al., 2017). Traditionally, antibiotics have been used for controlling the biofilms formation, whereas they faced a serious problem of antibiotic resistance, owing to the abuse of antibiotics. Many new methods for inhibiting the biofilms formation were explored. The antibacterial effect of graphene-based materials was firstly discovered by Hu et al. (Hu et al., 2010). Nowadays, more and more researchers have confirmed its effect in a various form.

Photodynamic therapy (PDT) is known as an alternative method for treatment of periodontitis and peri-implantitis. Pourhajibagher et al. investigated the effect of graphene quantum dot (GQD)–curcumin (Cur) on the perio-pathogen biofilms combined with PDT (Pourhajibagher et al., 2019). The production of reactive oxygen species (ROS) by GOD-Cur-PDT showed a dose-dependent tendency. In addition, the expression of *A. actinomycetemcomitans rcpA gene*, *P. gingivalis fimA gene*, and *P. intermedia inpA* gene was, respectively, reduced by 8.1-, 9.6-, and 11.8-fold. To improve the ICG photodynamic effect, Gholibegloo et al. fabricated the ICG-loaded GO, GO-Car, or GO-Car/HAp nanocomposites and investigated the antibacterial effect of *S. mutans* in planktonic forms and biofilms (Gholibegloo et al., 2018). Compared with the control group, GO, GO-Car, and GO-Car/HAp group decreased the survival of bacteria to 67%, 86.4%, and 78.2%, respectively. In preventing the biofilm formation of *S. mutans*, GO-Car group acquired the best effect. When treated with aPDT, the GO, GO-Car, and GO-Car/HAp group suppressed the biofilm formation up to 1.4%, 63.8%, and 56.8%, respectively. Then, the expression of *S. mutans gtfB* gene was decreased 6.0-, 9.0-, and 7.9-fold in GO, GO-Car, and GO-Car/HAp group, respectively, when without irradiation. Akbari et al. constructed the nano-GO (NGO)–ICG composite to enhance the efficiency of ICG (Akbari et al., 2017). The results showed that the NGO-ICG group achieved a significant decrease in the count of *E. faecalis* and significantly suppressed the formation of *E. faecalis* biofilms.

Besides, some researchers fabricated the graphene-based materials into GICs, PMMA, and dental adhesives to improve the physical properties and antibacterial ability (Bregnocchi et al., 2017; Lee J.-H. et al., 2018; Sun et al., 2018). Interestingly, Sun et al. evaluated the antibacterial effect of GIC/FG composites on *S. aureus* and *S. mutans*, showing that the highest antibacterial ability of FG (4 wt%) for *S. aureus* and *S. mutans* was 88.1% and 85.3%, respectively (Sun et al., 2018). Lee et al. constructed an nGO-PMMA and achieved a sustained antibacterial effect against *C. albicans* over 28 days (Lee SM. et al., 2018). Bregnocchi et al. added the GNP into the dental adhesive with different content and 0.2% GNP group, showing a significant decrease on the *S. mutans* (Bregnocchi et al., 2017).

Jin et al. studied the antibacterial effect of Ti-GO-Ag *in vitro* and *in vivo* (Jin et al., 2019). The GO group significantly suppressed the bacterial activity of *S. mutans* and *P. gingivalis* with the increasing concentration after 24 h. Zhao et al. confirmed the antibacterial activity of GO on *S. mutans* (Zhao et al., 2020). Ioannidis et al. synthesized the Ag-GO composites, showing obviously decreased bacterial activity (Ioannidis et al., 2019). Qiu et al. testified that GO had excellent antibacterial properties against *S. aureus* and *E. coli* (Jiajun et al., 2017). Potential mechanisms were further analyzed, including nanoknives, wrapping or trapping, and ROS production. rGO was also used as an antimicrobial agent in suppressing *S. mutans* by Wu et al. (Wu et al., 2018).

### Inhibition of the Growth of Fungal

Peri-implantitis is a common reason for the failure of dental implant. In addition, *Candida albicans* was found in the 31% peri-implantitis sites, which quickly attracted much attention (Schwarz et al., 2015). The species of *Candida albicans* in peri-implantitis patients were five times more than the health individuals (Alrabiah et al., 2019; Alsahhaf et al., 2019). Moreover, owing to the high resistant property of *Candida albicans*, the antifungal treatments are usually failure*.* The modification of dental implant coatings is a good method to prevent the formation of biofilms. Agarwalla et al. constructed a graphene nanocoating for twice (TiGD) and five times (TiGV) to evaluate the inhibition properties of *Candida albicans* biofilms (Agarwalla et al., 2020). According to XTT reduction assay, TiGD and TiGV group showed a lower absorbance compared with the control. Then, the colony-forming unit assay that displayed less viable yeast units on the TiGD and TiGV groups at all time points, indicating the inhibition effect of graphene on the fungal biofilm formations.

### Biosensor for Biomarker Detection From Saliva

Dental disease diagnosis can reduce the mortality rates of some serious diseases and improve the quality of life of patients. Owing to its superior electrical and mechanical ability, graphene-based materials are widely used on dental disease diagnosis (Goldoni et al., 2021).

#### Detection of Bacterial and Viral Markers

In 2012, Mannoor et al. made the first graphene nano-sensors on tooth enamel (Mannoor et al., 2012). They fabricated a graphene sensing element with wireless readout coil attached to the silk fibroin and then transferred onto tooth enamel. The specific biological recognition was acquired by self-assembling AMP-graphene peptides onto the graphene. The reduction of electrical resistance displayed the binding of a single *E. coli* on the bare graphene nanosensor. The AMP-modified graphene nanosensor that showed a strong connection between peptides and bacteria realized the detection and wireless remote monitoring of *Helicobacter pylori* in saliva. Gandouzi et al. build an electrochemical platform using rGO and gold nanoparticles, and the sensor showed high sensitivity to the markers (Islem et al., 2019). To diagnosis of periodontal disease in early stage, Lee et al. developed a sandwich-type biosensors to detect the human odontogenic ameloblast-associated protein (ODAM) (Wu et al., 2017). Checkin et al. fabricated an rGO/MoS_2_ glassy carbon electrode for detecting the human papillomavirus type 16 (HPV-16), showing the high stability and storage performance (Chekin et al., 2018).

#### Detection of Drugs

Saliva is a body fluid that could be used to monitor the drugs and other harmful substances. The biosensor is a good way to detect the analytes of drugs and harmful substances. Graphene-based materials are applying to fabricate the portable biosensors. For example, Mohamed and his coworkers constructed a bio-sensing platform for detecting two drugs: the benzocaine and the antipyrine. To increase the selectivity of biosensor, they decorated the GO sheets with metal nanoparticles, achieving the high reproducibility and good selectivity (Mohamed et al., 2017). Parate and his coworkers modified the electrochemical biosensors with graphene to monitor the byproducts of smoke and tobacco with a wide linear range of 1–100 nM and the sensitivity of 1.89 μA/decade (Parate et al., 2019).

#### Detection of Cancer Markers

Early diagnosis of disease is especially important for patients. Biomarker is a biological molecule which indicated the incidence of disease such as infections and cancers (Henry and Hayes, 2012). The overexpression of interleukin-8 (IL-8) has been reported to indicate the tumor progression in the oral cancer. Verma and his colleges fabricated a biosensor with rGO-modified ITO glass modified with rGO and then coated with Au NPs to detect IL-8 in saliva (Verma et al., 2017). The biosensor showed a high reproducibility and a long-term stability. After 3 months of dry storage, the retainment of biosensor is 94.3%. The performance of the biosensor was 91.8% even after 4 months of dry storage. To improve the performance of the biosensors, they also modified the GO with zinc oxide reduction and functionalized with IL-8 antibodies, showing that the sensitivity of the biosensor increased and the reproducibility was low with a RSD of 3.2% (Verma and Singh, 2019).

Owing to its excellent electrical properties, graphene-based appliances are reckoned as the gold standard in the biosensor fields. Nowadays, borophene has the similar anisotropic property with graphene (Tatullo et al., 2019b and Zavan et al., 2019). Song et al. had reported that, if the combination with borophene, graphene, and hydrogel in proper way, then it will improve the effect of implantable and wearable biosensors (Song et al., 2017). Therefore, when graphene combined with proper 2D nanomaterials, the efficiency of biosensors will be improved significantly. This may be also a good tendency in the development of graphene-based biosensors before the final clinical usage.

### Prevention of Enamel and Dentin From Demineralization

White spot lesion (WSL) is one of the most common side effects of orthodontic treatment, which is caused by enamel surface demineralization (Bishara and Ostby, 2008; Nam et al., 2019). Therefore, it is of great significance to overcome WSL in the process of orthodontic treatment. Nowadays, many researchers are focusing on the studying of new bonding agent composites to prevent enamel demineralization caused by bacteria. Owing to the prominent antibacterial activity of GO, Lee and his colleges added GO to a bioactive glass (BAG) (Lee J.-H. et al., 2018). With the increase of GO concentrations, the length of anti-demineralization of the GO group increased. Besides, GO-containing groups also showed superior antibacterial effect after 24 and 48 h. The anti-demineralization mechanism of the composites may be attributed to the synergic effect of antibacterial effect of GO and the ion-releasing effect of BAG. In conclusion, GO is a promising addition in the anti-demineralization of enamel in proper style.

Dental caries and dental erosion were associated with the demineralization of dentin, which caused by acids from bacteria, food, and environments, leading to dentin hypersensitivity and pain (Addy, 2005). Nizami et al. synthesized five different functionalized GO nanocomposites and evaluated the biological and prevention of demineralization effects (Nizami et al., 2020). Compared with the control, the dentin slices coated with GO-Ag, GO-Ag-CaF_2_, and GO-CaF_2_ all exhibited superior prevention of decalcification. Besides, GO-Ag and GO-Ag-CaF_2_ group showed better antibacterial activity compared with other groups, which may be related to the synergic effect of GO and Ag. Moreover, the color variation of f-GO coatings on the dentin surface is negligible, showing that GO is a promising dentin anti-demineralization resistant material.

### Collagen Membranes

COL membrane has been widely used in guided bone regeneration (GBR) and guided tissue regeneration (GTR) as barrier membrane to hinder the soft tissue invasion of new bone (Chu et al., 2017a, Deng, and Sun et al., 2017; Elgali et al., 2017; Wessing et al., 2018). Although COL membrane has many good properties such as facilitated manipulation and less surgical intervention, it still needs various modifications to improve the biocompatibility (Chu et al., 2017b, Deng, and Hou et al., 2017). Marco et al. enriched the COL membranes with GO *via* a non-covalent functionalization by the interaction between oxygenated carbon functional parts and COL through hydrogen bonding (Marco et al., 2017). The membranes enriched with GO displayed lower deformability, increased roughness, and higher stiffness. The stability of GO in the COL membranes was evaluated, and there was no obvious GO dissolution found in the bulk solution compared with the control. After cultured on membranes with 2 and 10 μg/ml GO for 3 days, the cell proliferations of hGFs were significantly higher than the control. With regard to the inflammatory response, the secretion of IL-6 and PGE2 showed significantly lower in cells cultured on the GO-coated membranes at day 3 compared with the control. When it comes to DPSCs, Radunovic et al. confirmed the superior cell proliferations on the GO-coated membranes at days 14 and 28 (Li et al., 2015). Meanwhile, the expression of BMP2 on the GO-coated membranes showed higher at days 3 and 7, whereas the expression of RUNX2 and SP7 on the GO-coated membranes showed augmented after 21 and 28 days when compared with the uncoated membrane. Moreover, LDH assay also confirmed that there was no cytotoxicity of GO coating on the COL membrane. Although GO displayed superior effect on the cell growth, osteogenic differentiation, and inflammation response on the COL membrane, more studies *in vitro* and *in vivo* also needed to testify their definitive effect.

### Drug Delivery

There is close interaction between bacteria and dental caries, and endodontic and periodontal diseases. Several groups of bacteria that require a common antibacterial strategy are usually involved. Amoxicillin (AMOX) is a kind of broad-spectrum antibiotic that is the first-choice antibiotic in the treatment of endodontic infection in Asian and European countries. In the conditional paste, the dose is not accurately controlled (Nan, 2016). Drug carrier can realize the gradual releasing of antibiotic drugs to easily achieve effective drug concentrations in the infected site. Trusek et al. found that GO had the potential in acting as a drug carrier especially in the therapy of dental inflammation (Trusek and Kijak, 2021). They linked the AMOX to GO using a peptide linker, which is Leu-Leu-Gly and then dispersed in the hydrogel. AMOX was released by enzymatic hydrolysis, showing the effective release of AMOX and the inhibition of bacteria strain growth.

## Challenges and Perspective

**FIGURE 9:**
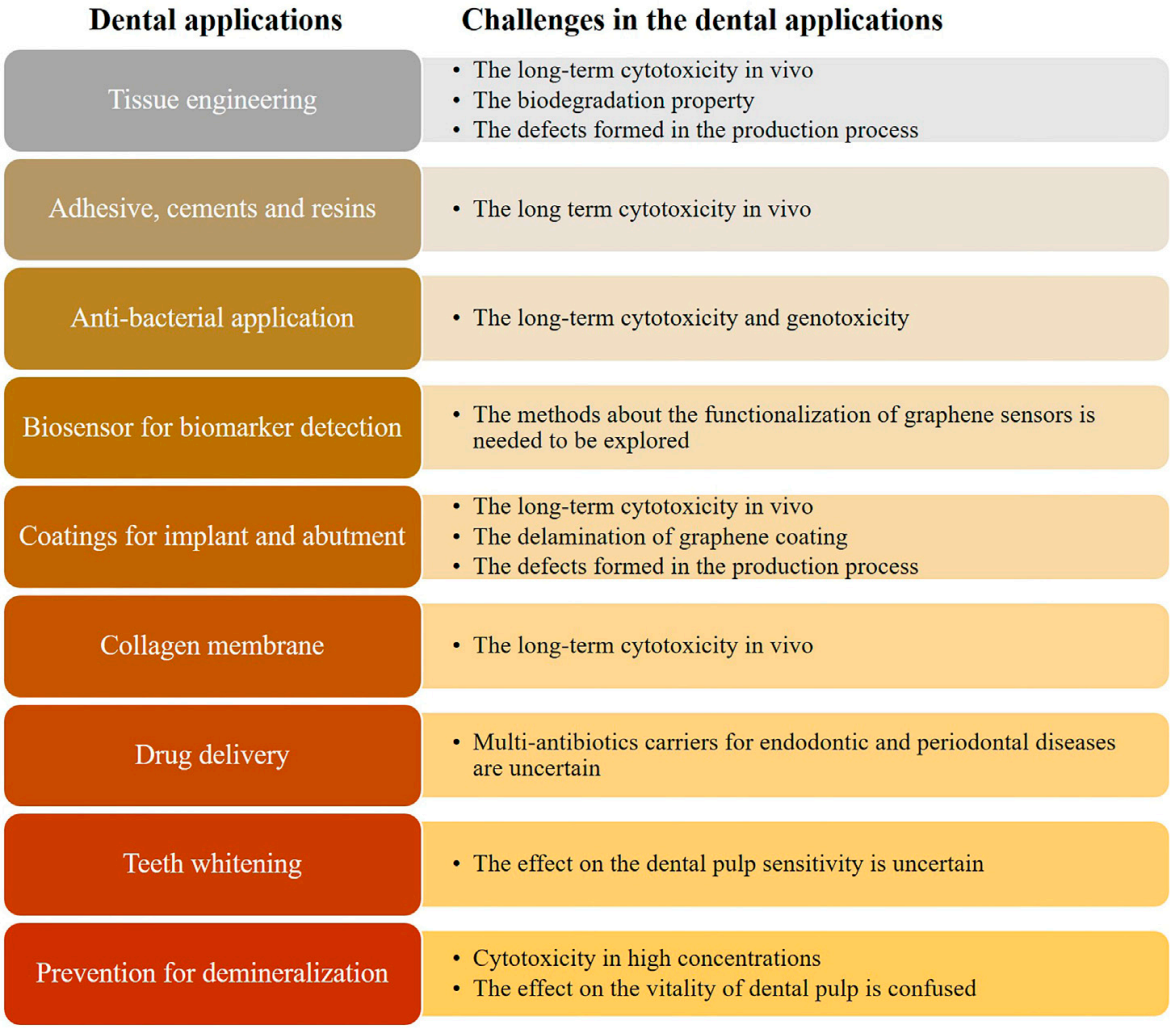
Challenges on the different applications in the dental fields.

Graphene-based materials, the promising candidate for dentistry materials, have been widely used in dentistry research, owing to its cell differentiation and antibacterial property. This review summarized recent advances in expanding the types of graphene-based materials and the studies about dentistry-related properties, deepening the understanding of categories of graphene-based materials. Compared with other related studies, it displayed a quite comprehensive and detailed review about the great achievements in dental applications such as bone tissue engineering, coatings for dental implants, antibacterial properties, and COL membranes. Except for the usage in the tissue engineering, dental implant coatings, COL membrane, and adhesive, we also focused on some new fields such as drug delivery, prevention of enamel and dentin from demineralization, biosensor for oral biomarker detection, and inhibition of the growth of fungal. For the application of graphene nanomaterials in the biosensor, it could be used to detect bacterial and viral markers, drugs, and cancer markers. With the development of the application of graphene in dentistry, there are still some challenges remaining to be tackled until the final commercialized (Figure 9).

### The Long-Term Cytotoxicity *in Vivo*

The excellent biomaterials should have a good biocompatibility without long-term cytotoxicity *in vitro* and *in vivo*. Because of the limited understanding of graphene and its derivatives, a major challenge in clinical applications is the uncertainty of its cytotoxicity *in vitro* and *in vivo* and its potential mechanisms. Up to date, there is not an agreement on the cytotoxicity and potential hazards of graphene-based materials according to various studies. The factors that involved in the cytotoxicity include concentrations, surface functionalization, types of graphene family and synthetic methods, and the layer numbers. Many studies have focused on the dose dependent effect on the cytotoxicity, yet there is still no agreement for the upper limit concentration (Duch et al., 2011). When it comes to the mechanisms of cytotoxicity, ROS may play a critical role in it. When it comes to the synthetic methods, graphene films with CVD method were reckoned to be biocompatible without obvious cytotoxicity. However, when the graphene is dispersed in solution, the cytotoxicity of cells may increase, which may be caused by the accumulation or the sharp-edge penetration into the cells. Therefore, we expect to see more and more studies for long-term biocompatibility *in vivo and in vitro*.

### The Method to Solve the Biodegradation

Another challenge of graphene-based materials is biodegradation, especially in the tissue engineering. With the formation of new tissue, the ideal biomaterial should be free of any toxic products. Up to date, there are limited literatures related to the biodegradation of graphene-based materials. Therefore, graphene-based material as an ideal biomaterial should be considered and explored to solve this problem.

### The Defects Formed During the Production Process

Although the graphene used in the current study is a controlled and uncontaminated defect-free sample, the synthesis quality still should be highly examined when it was used in the clinic. Actually, the formed variety of unpredictable defects is mainly due to the differences in the synthesis methods. Once the defects occurred, the properties such as susceptibility and electronic structure will be changed (Riccardo et al., 2018). Therefore, the study of controlling the formation of defects during the graphene synthesis would be a big challenge but rewarding research areas.

### Disturb the Cell Cycle

Little of the current studies are focused on the effect of GO on the cell cycle. Nowadays, Hashemi et al. innovatively focused on the effect of GO on the cell cycle (Hashemi et al., 2020). In the process of cell division, the synthesis of DNA is an important process. Certain mutagenic materials may cause the increased DNA synthesis in the S phase of cell cycle. In the study of Hashemi et al., GO increased the synthesis of DNA according to some underlying mechanisms such as damaged DNA, ROS production, and the double-strand breaks in the DNA. Cell apoptosis in mGO and nGO were greater, displaying a concentration and size-dependent effect. According to the result of cell cycle, there is a block occurred in the G_2_/M phase in the GO groups. Thus, the effect of GO on the cell cycle should be carefully considered and explored before the final clinical use.

### The Delamination of Graphene Coatings

Although graphene has many advantages in the dental field, its clinical translation still needs to be carefully considered. Graphene-based materials had been used as coatings mostly in tissue engineering and dental implants surfaces. When used as the dental implant coatings, friction could cause the delamination of carbon-based coatings on the titanium with the loads over 400 mN (Rosa et al., 2021). Rosa et al. reported that there was no significant difference between the solid rigid polyurethane (SRP) and the control following the integrity of graphene nanocoatings by stimulating in the SRP and pig maxilla. However, there was a 35% decrease in the coverage area in ROI C from the bone group. Therefore, the delamination of graphene should be considered carefully (Agarwalla et al., 2019; Ming et al., 2018). How to improve the bonding strength of graphene-based materials and substracts is a question needed to be carefully investigated. Up to date, the methods of graphene-based materials coatings on the titanium surfaces are mainly based on the physical methods such as spin-coating techniques and layer-by-layer self-assembly methods. There are still technical difficulties in the chemical combinations with graphene and titanium. Compared with the chemical methods, the physical combination is weak and unstable. Therefore, the delamination of graphene coatings is a risk factor when used as coatings. Some researchers had reported that titanium nanoparticles released from dental implants could cause the chronic inflammation on the soft and bone tissue around the dental implants. Therefore, when used graphene-based nanomaterials as coatings of dental implants, we should focus on the delamination of graphene coatings on the surrounding tissue inflammation.

### The Improved Methods About the Functionalization of Graphene Sensors

When used as a biosensor, the large surface of graphene-based nanomaterial provided good adhesive conformability and the functionalization of graphene could be achieved by AMP-graphene peptide for biological recognition. However, there are different kinds of bacteria in the mouth. Therefore, more strategies should be made to recognize more bacteria, and the methods for the functionalization of graphene biosensor are quite important.

### Single-Antibiotic Carrier for Endodontic and Periodontal Diseases

GO had been successfully used as a drug carrier for AMOX, which is a broad-spectrum antibiotic and could be used to control dental infections. However, the limitation is that whether two or more different drugs could be carried simultaneously is not sure and more studies are needed.

Eventually, graphene and its derivatives will be of great interest for a long time in the dental field. Although there are some limitations in the real clinical usage of dentistry, graphene, as a more reliable and more friendly biomaterial, can prompt more effective dental treatments in the near future.

### The Unfavorable Antibacterial Effect on the Polymicrobial Biofilms

Although there are many studies focused on the antibacterial effect of graphene-based materials on the single strain of bacteria or monoclonal biofilm, yet no study revealed the antibacterial effect on the mature polymicrobial biofilms. On the basis of the above limitations, there is still a long way before the final clinical usage of graphene-based materials in the dental fields.

## Conclusion

All acronyms and abbreviation used in the text are explained in Table 2. Just as we know, there are many advantages of graphene-based materials for the dental fields. However, many challenges also existed and need to be solved. All in all, we should confirm that the usage of graphene-based nanomaterials in dental fields deserved to be deeply studied and it can bring a quite new dental treatment concept in the near future.

**TABLE 2:** The abbreviations and full name used in this paper.

Abbreviations	Full name
WHO	World health organization
GO	Graphene oxide
rGO	Reduced graphene oxide
CVD	Chemical vapor deposition
DMF	N,N-dimethyl-form amide
NMP	N-methyl-2-pyrrolidone
DCB	Dichlorobenzene
BSA	Bovine serum albumin
N-Gr	Nitrogen-doped graphene
TRGO	Thermally reduced graphene oxide
BMSCs	Bone marrow mesenchymal stem cells
PDLCs	Periodontal ligament stem cells
DPSCs	Dental pulp stem cells
DFPCs	Dental follicle progenitor cells
RUNX2	Proteins runt-related transcription factor 2
OCN	Osteocalcin
COL	Collagen
hMSCs	Human mesenchymal stem cells
NFs	Nanofibers
PCL	Polycaprolactone
GICs	Glass ionomer cements
HAp	Hydroxyapatite
BCP	Biphasic calcium phosphate
EMFs	Electromagnetic fields
SCPAs	Stem cells of apical papilla
GNPs	Graphene nanoplatelets
FG	Fluorinated graphene
PMMA	Polymethyl methacrylate
nGO	Graphene oxide nanosheets
hGFs	Human gingival fibroblasts
hASCs	Human adipose-derived stem cells
hBMMSCs	Human bone marrow mesenchymal stem cells
Dex	Dexamethasone
MH	Minocycline hydrochloride
H2O2	Hydrogen peroxide
PDT	Photodynamic therapy
IL-8	Interleukin-8
WSL	White spot lesion
BAG	Bioactive glass
GBR	Guided bone regeneration
GTR	Guided tissue regeneration
AMOX	Amoxicillin
SRP	Solid rigid polyurethane

## References

[B1] Addy M. (2005). Tooth Brushing, Tooth Wear and Dentine Hypersensitivity - Are They Associated? Int. Dental J. 55 (4 Suppl. 1), 261–267. 10.1111/j.1875-595x.2005.tb00063.x 16167604

[B2] Agarwalla S. V. Ellepola K. Costa M. C. F. d. Fechine G. J. M. Morin J. L. P. Castro Neto A. H. (2019). Hydrophobicity of Graphene as a Driving Force for Inhibiting Biofilm Formation of Pathogenic Bacteria and Fungi. Dental Mater. 35 (3), 403–413. 10.1016/j.dental.2018.09.016 30679015

[B3] Agarwalla S. V. Ellepola K. Silikas N. Castro Neto A. Seneviratne C. J. Rosa V. (2021). Persistent Inhibition of Candida Albicans Biofilm and Hyphae Growth on Titanium by Graphene Nanocoating. Dental Mater. 37 (2), 370–377. 10.1016/j.dental.2020.11.028 33358443

[B4] Akbari T. Pourhajibagher M. Hosseini F. Chiniforush N. Gholibegloo E. Khoobi M. (2017). The Effect of Indocyanine green Loaded on a Novel Nano-Graphene Oxide for High Performance of Photodynamic Therapy against *Enterococcus faecalis* . Photodiagnosis Photodynamic Ther. 20, 148–153. 10.1016/j.pdpdt.2017.08.017 28867453

[B5] Alp G. Johnston W. M. Yilmaz B. (2019). Optical Properties and Surface Roughness of Prepolymerized Poly(methyl Methacrylate) Denture Base Materials. The J. Prosthetic Dentistry 121 (2), 347–352. 10.1016/j.prosdent.2018.03.001 30143239

[B6] Alrabiah M. Alshagroud R. S. Alsahhaf A. Almojaly S. A. Abduljabbar T. Javed F. (2019). Presence of Candida Species in the Subgingival Oral Biofilm of Patients with Peri‐implantitis. Clin. Implant Dent Relat. Res. 21 (4), 781–785. 10.1111/cid.12760 30908836

[B7] Alsahhaf A. Al‐Aali K. A. Alshagroud R. S. Alshiddi I. F. Alrahlah A. Abduljabbar T. (2019). Comparison of Yeast Species in the Subgingival Oral Biofilm of Individuals with Type 2 Diabetes and Peri‐implantitis and Individuals with Peri‐implantitis without Diabetes. J. Periodontol. 90 (12), 1383–1389. 10.1002/JPER.19-0091 31318043

[B8] Amiryaghoubi N. Noroozi Pesyan N. Fathi M. Omidi Y. (2020). Injectable Thermosensitive Hybrid Hydrogel Containing Graphene Oxide and Chitosan as Dental Pulp Stem Cells Scaffold for Bone Tissue Engineering. Int. J. Biol. Macromolecules 162, 1338–1357. 10.1016/j.ijbiomac.2020.06.138 32561280

[B9] Ansari M. O. Gauthaman K. Essa A. Bencherif S. A. Memic A. (2019). Graphene and Graphene-Based Materials in Biomedical Applications. Cmc 26 (38), 6834–6850. 10.2174/0929867326666190705155854 31284851

[B10] Azadian E. Arjmand B. Ardeshirylajimi A. Hosseinzadeh S. Omidi M. Khojasteh A. (2020). Polyvinyl Alcohol Modified Polyvinylidene Fluoride‐graphene Oxide Scaffold Promotes Osteogenic Differentiation Potential of Human Induced Pluripotent Stem Cells. J. Cel. Biochem. 121 (5-6), 3185–3196. 10.1002/jcb.29585 31886565

[B11] Azevedo L. Antonaya-Martin J. Molinero-Mourelle P. del Rio-Highsmith J. (2019). Improving PMMA Resin Using Graphene Oxide for a Definitive Prosthodontic Rehabilitation - a Clinical Report. J. Clin. Exp. Dent 11, 670–674. 10.4317/jced.55883 PMC6730997 31516667

[B12] Bacali C. Badea M. Moldovan M. Sarosi C. Nastase V. Baldea I. (2019). The Influence of Graphene in Improvement of Physico-Mechanical Properties in PMMA Denture Base Resins. Materials 12 (14), 2335. 10.3390/ma12142335 PMC6678796 31340462

[B13] Bacali C. Baldea I. Moldovan M. Carpa R. Olteanu D. E. Filip G. A. (2020). Flexural Strength, Biocompatibility, and Antimicrobial Activity of a Polymethyl Methacrylate Denture Resin Enhanced with Graphene and Silver Nanoparticles. Clin. Oral Invest. 24 (4), 2713–2725. 10.1007/s00784-019-03133-2 31734793

[B14] Barfeie A. Wilson J. Rees J. (2015). Implant Surface Characteristics and Their Effect on Osseointegration. Br. Dent J. 218 (5), E9. 10.1038/sj.bdj.2015.171 25766196

[B15] Bei H. Yang Y. Zhang Q. Tian Y. Luo X. Yang M. (2019). Graphene-Based Nanocomposites for Neural Tissue Engineering. Molecules 24 (4), 658. 10.3390/molecules24040658 PMC6413135 30781759

[B16] Berglundh T. Jepsen S. Stadlinger B. Terheyden H. (2019). Peri-implantitis and its Prevention. Clin. Oral Impl Res. 30 (2), 150–155. 10.1111/clr.13401 30636066

[B17] Bishara S. E. Ostby A. W. (2008). White Spot Lesions: Formation, Prevention, and Treatment. Semin. Orthod. 14 (3), 174–182. 10.1053/j.sodo.2008.03.002

[B18] Bregnocchi A. Zanni E. Uccelletti D. Marra F. Cavallini D. De Angelis F. (2017). Graphene-based Dental Adhesive with Anti-biofilm Activity. J. Nanobiotechnol. 15 (1), 89. 10.1186/s12951-017-0322-1 PMC5728064 29233187

[B19] Bressan E. Ferroni L. Gardin C. Bellin G. Sbricoli L. Sivolella S. (2019). Metal Nanoparticles Released from Dental Implant Surfaces: Potential Contribution to Chronic Inflammation and Peri-Implant Bone Loss. Materials 12 (12), 2036. 10.3390/ma12122036 PMC6630980 31242601

[B20] Carey C. M. Clifton M. (2014). Tooth Whitening: What We Now Know. J. Evid. Based Dental Pract. 14, 70–76. 10.1016/j.jebdp.2014.02.006 PMC4058574 24929591

[B21] Chekin F. Bagga K. Subramanian P. Jijie R. Singh S. K. Kurungot S. (2018). Nucleic Aptamer Modified Porous Reduced Graphene oxide/MoS2 Based Electrodes for Viral Detection: Application to Human Papillomavirus (HPV). Sensors Actuators B: Chem. 262, 991–1000. 10.1016/j.snb.2018.02.065

[B22] Cherian R. S. Sandeman S. Ray S. Savina I. N. J. A. P.V. M. (2019). Green Synthesis of Pluronic Stabilized Reduced Graphene Oxide: Chemical and Biological Characterization. Colloids Surf. B: Biointerfaces 179, 94–106. 10.1016/j.colsurfb.2019.03.043 30952020

[B23] Cho B. H. Ko W. B. (2013). Preparation of Graphene-ZrO2 Nanocomposites by Heat Treatment and Photocatalytic Degradation of Organic Dyes. J. Nanosci. Nanotech. 13 (11), 7625–7630. 10.1166/jnn.2013.7819 24245304

[B24] Chouirfa H. Bouloussa H. Migonney V. Falentin-Daudré C. (2019). Review of Titanium Surface Modification Techniques and Coatings for Antibacterial Applications. Acta Biomater. 83, 37–54. 10.1016/j.actbio.2018.10.036 30541702

[B25] Chu C. Deng J. Hou Y. Xiang L. Wu Y. Qu Y. (2017a). Application of PEG and EGCG Modified Collagen-Base Membrane to Promote Osteoblasts Proliferation. Mater. Sci. Eng. C 76, 31–36. 10.1016/j.msec.2017.02.157 28482532

[B26] Chu C. Deng J. Sun X. Qu Y. Man Y. (2017b). Collagen Membrane and Immune Response in Guided Bone Regeneration: Recent Progress and Perspectives. Tissue Eng. B: Rev. 23 (5), 421–435. 10.1089/ten.teb.2016.0463 28372518

[B27] Cote L. J. Cruz-Silva R. Huang J. (2009). Flash Reduction and Patterning of Graphite Oxide and its Polymer Composite. J. Am. Chem. Soc. 131 (31), 11027–11032. 10.1021/ja902348k 19601624

[B29] D’Onofrio N. Balestrieri A. Neglia G. Monaco A. Tatullo M. Casale R. (2019). Antioxidant and Anti-inflammatory Activities of Buffalo Milk δ-Valerobetaine. J. Agric. Food Chem. 67 (6), 1702–1710. 10.1021/acs.jafc.8b07166 30661355

[B30] Du Z. Wang C. Zhang R. Wang X. Li X. (2020). Applications of Graphene and its Derivatives in Bone Repair: Advantages for Promoting Bone Formation and Providing Real-Time Detection, Challenges and Future Prospects. Ijn 15, 7523–7551. 10.2147/IJN.S271917 33116486 PMC7547809

[B31] Dubey N. Rajan S. S. Bello Y. D. Min K.-S. Rosa V. (2017). Graphene Nanosheets to Improve Physico-Mechanical Properties of Bioactive Calcium Silicate Cements. Materials 10 (6), 606. 10.3390/ma10060606 PMC5553423 28772959

[B32] Duch M. C. Budinger G. R. S. Liang Y. T. Soberanes S. Urich D. Chiarella S. E. (2011). Minimizing Oxidation and Stable Nanoscale Dispersion Improves the Biocompatibility of Graphene in the Lung. Nano Lett. 11 (12), 5201–5207. 10.1021/nl202515a 22023654 PMC3237757

[B33] Dybowska-Sarapuk Ł. Kotela A. Krzemiński J. Wróblewska M. Marchel H. Romaniec M. (2017). Graphene Nanolayers as a New Method for Bacterial Biofilm Prevention: Preliminary Results. J. Aoac Int. 100 (4), 900–904. 10.5740/jaoacint.17-0164 28623661

[B34] El Bahra S. Ludwig K. Samran A. Freitag-Wolf S. Kern M. (2013). Linear and Volumetric Dimensional Changes of Injection-Molded PMMA Denture Base Resins. Dental Mater. 29 (11), 1091–1097. 10.1016/j.dental.2013.07.020 24001949

[B35] Elgali I. Omar O. Dahlin C. Thomsen P. (2017). Guided Bone Regeneration: Materials and Biological Mechanisms Revisited. Eur. J. Oral Sci. 125 (5), 315–337. 10.1111/eos.12364 28833567 PMC5601292

[B36] Fallahzadeh F. Safarzadeh-Khosroshahi S. Atai M. (2017). Dentin Bonding Agent with Improved Bond Strength to Dentin through Incorporation of Sepiolite Nanoparticles. J. Clin. Exp. Dent 9 (6), e738–e742. 10.4317/jced.53722 28638548 PMC5474327

[B37] Fan Z. Wang J. Wang Z. Ran H. Li Y. Niu L. (2014). One-pot Synthesis of Graphene/hydroxyapatite Nanorod Composite for Tissue Engineering. Carbon 66, 407–416. 10.1016/j.carbon.2013.09.016

[B38] Farooq I. Ali S. Al-Saleh S. AlHamdan E. M. AlRefeai M. H. Abduljabbar T. (2021). Synergistic Effect of Bioactive Inorganic Fillers in Enhancing Properties of Dentin Adhesives-A Review. Polymers 13 (13), 2169. 10.3390/polym13132169 34209016 PMC8271823

[B39] Gandouzi I. Tertis M. Cernat A. Saidane-Mosbahi D. Ilea A. Cristea C. (2019). A Nanocomposite Based on Reduced Graphene and Gold Nanoparticles for Highly Sensitive Electrochemical Detection of pseudomonas Aeruginosa through its Virulence Factors. Materials 12 (7), 1180. 10.3390/ma12071180 PMC6480001 30978921

[B40] Gholibegloo E. Karbasi A. Pourhajibagher M. Chiniforush N. Ramazani A. Akbari T. (2018). Carnosine-graphene Oxide Conjugates Decorated with Hydroxyapatite as Promising Nanocarrier for ICG Loading with Enhanced Antibacterial Effects in Photodynamic Therapy against Streptococcus Mutans. J. Photochem. Photobiol. B: Biol. 181, 14–22. 10.1016/j.jphotobiol.2018.02.004 29482032

[B41] Ghuge A. D. Shirode A. R. Kadam V. J. (2017). Graphene: A Comprehensive Review. Cdt 18 (6), 724–733. 10.2174/1389450117666160709023425 27397067

[B42] Goldoni R. Farronato M. Connelly S. T. Tartaglia G. M. Yeo W.-H. (2021). Recent Advances in Graphene-Based Nanobiosensors for Salivary Biomarker Detection. Biosens. Bioelectron. 171, 112723. 10.1016/j.bios.2020.112723 33096432 PMC7666013

[B43] Gordon S. C. Donoff R. B. (2016). Problems and Solutions for Interprofessional Education in north American Dental Schools. Dental Clin. North America 60 (4), 811–824. 10.1016/j.cden.2016.05.002 27671955

[B44] Gu M. Liu Y. Chen T. Du F. Zhao X. Xiong C. (2014). Is Graphene a Promising Nano-Material for Promoting Surface Modification of Implants or Scaffold Materials in Bone Tissue Engineering? Tissue Eng. Part B: Rev. 20 (5), 477–491. 10.1089/ten.TEB.2013.0638 24447041 PMC4186769

[B46] Guazzo R. Gardin C. Bellin G. Sbricoli L. Ferroni L. Ludovichetti F. (2018). Graphene-Based Nanomaterials for Tissue Engineering in the Dental Field. Nanomaterials 8 (5), 349. 10.3390/nano8050349 PMC5977363 29783786

[B47] Guo H.-L. Wang X.-F. Qian Q.-Y. Wang F.-B. Xia X.-H. (2009). A green Approach to the Synthesis of Graphene Nanosheets. ACS Nano 3 (9), 2653–2659. 10.1021/nn900227d 19691285

[B48] Hashemi E. Akhavan O. Shamsara M. Ansari Majd S. Sanati M. H. Daliri Joupari M. (2020). Graphene Oxide Negatively Regulates Cell Cycle in Embryonic Fibroblast Cells. Ijn 15, 6201–6209. 10.2147/IJN.S260228 32884270 PMC7443459

[B49] He J. Zhu X. Qi Z. Wang C. Mao X. Zhu C. (2015). Killing Dental Pathogens Using Antibacterial Graphene Oxide. ACS Appl. Mater. Inter. 7 (9), 5605–5611. 10.1021/acsami.5b01069 25705785

[B50] He Q. Wu S. Gao S. Cao X. Yin Z. Li H. (2011). Transparent, Flexible, All-Reduced Graphene Oxide Thin Film Transistors. ACS Nano 5 (6), 5038–5044. 10.1021/nn201118c 21524119

[B51] Henry N. L. Hayes D. F. (2012). Cancer Biomarkers. Mol. Oncol. 6 (2), 140–146. 10.1016/j.molonc.2012.01.010 22356776 PMC5528374

[B52] Hideaki Hirooka Stefan Renvert (2019). Diagnosis of Periimplant Disease. Implant Dent 28 (2), 144–149. 10.1097/ID.0000000000000868 30807404

[B53] Hu W. Peng C. Luo W. Lv M. Li X. Li D. (2010). Graphene-based Antibacterial Paper. ACS Nano 4 (7), 4317–4323. 10.1021/nn101097v 20593851

[B54] Huang X. Qi X. Boey F. Zhang H. (2012). Graphene-based Composites. Chem. Soc. Rev. 41 (2), 666–686. 10.1039/c1cs15078b 21796314

[B55] Hummers W. S. Offeman R. E. (1958). Preparation of Graphitic Oxide. J. Am. Chem. Soc. 208, 1334–1339. 10.1021/ja01539a017

[B130] I-Hsuan Yu-Pin Lee Chen-Fu Wang Lai-Hao (2016). Evaluating a Cobalt-Tetraphenylporphyrin Complex, Functionalized with a Reduced Graphene Oxide Nanocomposite, for Improved Tooth Whitening. J. Esthet Restor Dent 28 (5), 321–329. 10.1111/jerd.12240 27530080

[B56] Ioannidis K. Niazi S. Mylonas P. Mannocci F. Deb S. (2019). The Synthesis of Nano Silver-Graphene Oxide System and its Efficacy against Endodontic Biofilms Using a Novel Tooth Model. Dental Mater. 35 (11), 1614–1629. 10.1016/j.dental.2019.08.105 31530433

[B124] Jelena S. Bosko T. Nadja N. Jasna V. Radmila P. Rados G. (2018). Differentiation of Stem Cells from Apical Papilla into Neural Lineage Using Graphene Dispersion and Single Walled Carbon Nanotubes. J. Biomed. Mater. Res. 106 (10), 2653–2661. 10.1002/jbm.a.36461 29896770

[B57] Jeong W.-S. Kwon J.-S. Lee J.-H. Uhm S.-H. Ha Choi E. Kim K.-M. (2017). Bacterial Attachment on Titanium Surfaces Is Dependent on Topography and Chemical Changes Induced by Nonthermal Atmospheric Pressure Plasma. Biomed. Mater. 12 (4), 045015. 10.1088/1748-605X/aa734e 28746053

[B58] Jin J. Fei D. Zhang Y. Wang Q. (2019). Functionalized Titanium Implant in Regulating Bacteria and Cell Response. Ijn 14, 1433–1450. 10.2147/IJN.S193176 30863070 PMC6390868

[B59] Jin J. Zhang L. Shi M. Zhang Y. Wang Q. (2017). Ti-GO-Ag Nanocomposite: The Effect of Content Level on the Antimicrobial Activity and Cytotoxicity. Ijn 12, 4209–4224. 10.2147/IJN.S134843 28652728 PMC5473600

[B60] Jung H. S. Lee T. Kwon I. K. Kim H. S. Hahn S. K. Lee C. S. (2015). Surface Modification of Multipass Caliber-Rolled Ti alloy with Dexamethasone-Loaded Graphene for Dental applicationsResearch Support, Non-U.S. Gov't]. ACS Appl. Mater. Interfacesacs Appl. Mater. Inter. 7 (18), 9598–9607. 10.1021/acsami.5b03431 25909563

[B62] Kazemizadeh F. Malekfar R. (2018). One Step Synthesis of Porous Graphene by Laser Ablation: A New and Facile Approach. Physica B: Condensed Matter 530, 236–241. 10.1016/j.physb.2017.11.052

[B63] Khan A. A. Al‐Khureif A. A. Saadaldin S. A. Mohamed B. A. Musaibah A. S. O. Divakar D. D. (2019). Graphene Oxide‐based Experimental Silane Primers Enhance Shear Bond Strength between Resin Composite and Zirconia. Eur. J. Oral Sci. 127 (6), 570–576. 10.1111/eos.12665 31823433

[B64] Kim J.-W. Shin Y. Lee J.-J. Bae E.-B. Jeon Y.-C. Jeong C.-M. (2017). The Effect of Reduced Graphene Oxide-Coated Biphasic Calcium Phosphate Bone Graft Material on Osteogenesis. Ijms 18 (8), 1725. 10.3390/ijms18081725 PMC5578115 28786931

[B61] Kohei K. Hirofumi M. Erika N. Saori M. Akihito K. Akito T. (2018). Characterization and Evaluation of Graphene Oxide Scaffold for Periodontal Wound Healing of Class II Furcation Defects in Dog. Int. J. Nanomed. 13, 2365–2376. 10.2147/IJN.S163206 PMC5912619 29713167

[B65] Koldsland O. C. Wohlfahrt J. C. Aass A. M. (2018). Surgical Treatment of Peri-Implantitis: Prognostic Indicators of Short-Term Results. J. Clin. Periodontol. 45 (1), 100–113. 10.1111/jcpe.12816 28902415

[B66] Kordbacheh C. K. Finkelstein J. Papapanou P. N. (2019). Peri‐implantitis Prevalence, Incidence Rate, and Risk Factors: A Study of Electronic Health Records at a U.S. Dental School. Clin. Oral Impl Res. 30 (4), 306–314. 10.1111/clr.13416 30768875

[B67] Kovtyukhova N. I. Ollivier P. J. Martin B. R. Mallouk T. E. Chizhik S. A. Buzaneva E. V. (1999). Layer-by-Layer Assembly of Ultrathin Composite Films from Micron-Sized Graphite Oxide Sheets and Polycations. Chem. Mater. 11 (3), 771–778. 10.1021/cm981085u

[B68] Kulshrestha S. Khan S. Meena R. Singh B. R. Khan A. U. (2014). A Graphene/zinc Oxide Nanocomposite Film Protects Dental Implant Surfaces against cariogenicStreptococcus Mutans. Biofouling 30 (9-10), 1281–1294. 10.1080/08927014.2014.983093 25431994

[B69] Kwon S. R. Wertz P. W. (2015). Review of the Mechanism of Tooth Whitening. J. Esthet Restor Dent 27 (5), 240–257. 10.1111/jerd.12152 25969131

[B70] Lamster I. B. (2021). The 2021 WHO Resolution on Oral Health. Int. Dental J. 71 (4), 279–280. 10.1016/j.identj.2021.06.003 PMC9275116 34256923

[B71] Lazauskas A. Marcinauskas L. Andrulevicius M. (2018). Photothermal Reduction of Thick Graphene Oxide Multilayer Films via Direct Laser Writing: Morphology, Structural and Chemical Properties. Superlattices and Microstructures 122, 36–45. 10.1016/j.spmi.2018.08.024

[B72] Lee J.-H. Jo J.-K. Kim D.-A. Patel K. D. Kim H.-W. Lee H.-H. (2018a). Nano-graphene Oxide Incorporated into PMMA Resin to Prevent Microbial Adhesion. Dental Mater. 34 (4), e63–e72. 10.1016/j.dental.2018.01.019 29402540

[B73] Lee J. H. Shin Y. C. Jin O. S. Kang S. H. Hwang Y.-S. Park J.-C. (2015a). Reduced Graphene Oxide-Coated Hydroxyapatite Composites Stimulate Spontaneous Osteogenic Differentiation of Human Mesenchymal Stem Cells. Nanoscale 7 (27), 11642–11651. 10.1039/c5nr01580d 26098486

[B74] Lee J. H. Shin Y. C. Lee S.-M. Jin O. S. Kang S. H. Hong S. W. (2015b). Enhanced Osteogenesis by Reduced Graphene Oxide/Hydroxyapatite Nanocomposites. Sci. Rep. 5, 18833. 10.1038/srep18833 26685901 PMC4685392

[B75] Lee S. M. Yoo S. Y. Kim I. R. Park B. S. Son W. S. Ko C. C. (2018b). Enamel Anti-demineralization Effect of Orthodontic Adhesive Containing Bioactive Glass and Graphene Oxide: An *In-Vitro* Study. Materials (Basel) 11 (9), 1728. 10.3390/ma11091728 PMC6163975 30223468

[B76] Li H. Xie Y. Li K. Huang L. Huang S. Zhao B. (2014). Microstructure and Wear Behavior of Graphene Nanosheets-Reinforced Zirconia Coating. Ceramics Int. 40 (8), 12821–12829. 10.1016/j.ceramint.2014.04.136

[B77] Li J. Wang G. Geng H. Zhu H. Zhang M. Di Z. (2015). CVD Growth of Graphene on NiTi alloy for Enhanced Biological Activity. ACS Appl. Mater. Inter. 7 (36), 19876–19881. 10.1021/acsami.5b06639 26323051

[B78] Li Q.-L. Huang N. Chen J. Wan G. Zhao A. Chen J. (2009). Anticoagulant Surface Modification of Titanium via Layer-By-Layer Assembly of Collagen and Sulfated Chitosan Multilayers. J. Biomed. Mater. Res. 89A (3), 575–584. 10.1002/jbm.a.31999 18435411

[B79] Li Z. Yao Y. Lin Z. Moon K.-S. Lin W. Wong C. (2010). Ultrafast, Dry Microwave Synthesis of Graphene Sheets. J. Mater. Chem. 20 (23), 4781. 10.1039/c0jm00168f

[B80] Liao C. Li Y. Tjong S. (2018). Graphene Nanomaterials: Synthesis, Biocompatibility, and Cytotoxicity. Ijms 19 (11), 3564. 10.3390/ijms19113564 PMC6274822 30424535

[B81] Lim K.-T. Seonwoo H. Choi K. S. Jin H. Jang K.-J. Kim J. (2016). Pulsed-Electromagnetic-Field-Assisted Reduced Graphene Oxide Substrates for Multidifferentiation of Human Mesenchymal Stem Cells. Adv. Healthc. Mater. 5 (16), 2069–2079. 10.1002/adhm.201600429 27332788

[B82] Liu J. Fu S. Yuan B. Li Y. Deng Z. (2010). Toward a Universal "adhesive Nanosheet" for the Assembly of Multiple Nanoparticles Based on a Protein-Induced Reduction/Decoration of Graphene Oxide. J. Am. Chem. Soc. 132 (21), 7279–7281. 10.1021/ja100938r 20462190

[B83] Liu L.-N. Zhang X.-H. Liu H.-H. Li K.-H. Wu Q.-H. Liu Y. (2020). Osteogenesis Differences Around Titanium Implant and in Bone Defect between Jaw Bones and Long Bones. J. Craniofac. Surg. 31 (8), 2193–2198. 10.1097/SCS.0000000000006795 33136853

[B84] Liu N. Luo F. Wu H. Liu Y. Zhang C. Chen J. (2008). One-Step Ionic-Liquid-Assisted Electrochemical Synthesis of Ionic-Liquid-Functionalized Graphene Sheets Directly from Graphite. Adv. Funct. Mater. 18, 1518–1525. 10.1002/adfm.200700797

[B85] Liu X. Miller A. L. Park S. George M. N. Waletzki B. E. Xu H. (2019). Two-Dimensional Black Phosphorus and Graphene Oxide Nanosheets Synergistically Enhance Cell Proliferation and Osteogenesis on 3D Printed Scaffolds. ACS Appl. Mater. Inter. 11 (26), 23558–23572. 10.1021/acsami.9b04121 PMC8942345 31199116

[B86] Lu J. Sun J. Zou D. Song J. Yang S. (2020). Graphene-Modified Titanium Surface Enhances Local Growth Factor Adsorption and Promotes Osteogenic Differentiation of Bone Marrow Stromal Cells. Front. Bioeng. Biotechnol. 8, 621788. 10.3389/fbioe.2020.621788 33511107 PMC7835422

[B87] Mannoor M. S. Tao H. Clayton J. D. Sengupta A. Kaplan D. L. Naik R. R. (2012). Graphene-based Wireless Bacteria Detection on Tooth Enamel. Nat. Commun. 3, 763. 10.1038/ncomms1767 22453836

[B88] Mao S. Lu G. Yu K. Bo Z. Chen J. (2010). Specific Protein Detection Using Thermally Reduced Graphene Oxide Sheet Decorated with Gold Nanoparticle-Antibody Conjugates. Adv. Mater. 22 (32), 3521–3526. 10.1002/adma.201000520 20665564

[B89] Marcano D. C. Kosynkin D. V. Berlin J. M. Sinitskii A. Sun Z. Slesarev A. (2010). Improved Synthesis of Graphene Oxide. ACS Nano 4 (8), 4806–4814. 10.1021/nn1006368 20731455

[B28] Marco P. D. Zara S. Colli M. D. Radunovic M. Lazović V. Ettorre V. (2017). Graphene Oxide Improves the Biocompatibility of Collagen Membranes in an *In Vitro* Model of Human Primary Gingival Fibroblasts. Biomed. Mater. 12 (5), 055005. 10.1088/1748-605X/aa7907 28607223

[B90] Matsuo H. Suenaga H. Takahashi M. Suzuki O. Sasaki K. Takahashi N. (2015). Deterioration of Polymethyl Methacrylate Dentures in the Oral Cavity. Dent. Mater. J. 34 (2), 234–239. 10.4012/dmj.2014-089 25740307

[B91] Miyaji H. Kato A. Takita H. Iwanaga T. Momose T. Ogawa K. (2016). Graphene Oxide Scaffold Accelerates Cellular Proliferative Response and Alveolar Bone Healing of Tooth Extraction Socket. Ijn 11, 2265–2277. 10.2147/IJN.S104778 27307729 PMC4887064

[B45] Ming G. Lv L. Feng D. Niu T. Tong C. Xia D. (2018). Effects of thermal Treatment on the Adhesion Strength and Osteoinductive Activity of Single-Layer Graphene Sheets on Titanium Substrates. Sci. Rep. 8 (1), 8141. 10.1038/s41598-018-26551-w 29802306 PMC5970187

[B92] Mohamed M. A. Atty S. A. Merey H. A. Fattah T. A. Foster C. W. Banks C. E. (2017). Titanium Nanoparticles (TiO2)/graphene Oxide Nanosheets (GO): An Electrochemical Sensing Platform for the Sensitive and Simultaneous Determination of Benzocaine in the Presence of Antipyrine. Analyst 142 (19), 3674–3679. 10.1039/c7an01101f 28836639

[B93] Nam H.-J. Kim Y.-M. Kwon Y. H. Kim I.-R. Park B.-S. Son W.-S. (2019). Enamel Surface Remineralization Effect by Fluorinated Graphite and Bioactive Glass-Containing Orthodontic Bonding Resin. Materials 12 (8), 1308. 10.3390/ma12081308 PMC6515273 31013602

[B94] Nan A. (2016). Miscellaneous Drugs, Materials, Medical Devices and Techniques. Side Effects Drugs Annu. 38, 523–532. 10.1016/bs.seda.2016.09.002

[B95] Nizami M. Z. I. Nishina Y. Yamamoto T. Shinoda-Ito Y. Takashiba S. (2020). Functionalized Graphene Oxide Shields Tooth Dentin from Decalcification. J. Dent. Res. 99 (2), 182–188. 10.1177/0022034519894583 31860805

[B96] Norahan M. H. Amroon M. Ghahremanzadeh R. Rabiee N. Baheiraei N. (2019). Reduced Graphene Oxide: Osteogenic Potential for Bone Tissue Engineering. IET nanobiotechnol. 13 (7), 720–725. 10.1049/iet-nbt.2019.0125 31573541 PMC8676151

[B97] Norimatsu W. Kusunoki M. (2014). Epitaxial Graphene on SiC{0001}: Advances and Perspectives. Phys. Chem. Chem. Phys. 16 (8), 3501–3511. 10.1039/c3cp54523g 24434866

[B98] Novoselov K. S. Geim A. K. Morozov S. V. Jiang D. Zhang Y. Dubonos S. V. (2004). Electric Field Effect in Atomically Thin Carbon Films. Science 306 (5696), 666–669. 10.1126/science.1102896 15499015

[B99] Olteanu D. Filip A. Socaci C. Biris A. R. Filip X. Coros M. (2015). Cytotoxicity Assessment of Graphene-Based Nanomaterials on Human Dental Follicle Stem Cells. Colloids Surf. B: Biointerfaces 136, 791–798. 10.1016/j.colsurfb.2015.10.023 26529387

[B100] Parate K. Karunakaran C. Claussen J. C. (2019). Electrochemical Cotinine Sensing with a Molecularly Imprinted Polymer on a Graphene-Platinum Nanoparticle Modified Carbon Electrode towards Cigarette Smoke Exposure Monitoring. Sensors Actuators B: Chem. 287, 165–172. 10.1016/j.snb.2019.02.032

[B101] Park C. Park S. Lee D. Choi K. S. Lim H.-P. Kim J. (2017). Graphene as an Enabling Strategy for Dental Implant and Tissue Regeneration. Tissue Eng. Regen. Med. 14 (5), 481–493. 10.1007/s13770-017-0052-3 30603503 PMC6171627

[B102] Park S. Ruoff R. S. (2009). Chemical Methods for the Production of Graphenes. Nat. Nanotech 4 (4), 217–224. 10.1038/nnano.2009.58 19350030

[B103] Parnia F. Yazdani J. Javaherzadeh V. Maleki Dizaj S. (2017). Overview of Nanoparticle Coating of Dental Implants for Enhanced Osseointegration and Antimicrobial Purposes. J. Pharm. Pharm. Sci. 20 (0), 148–160. 10.18433/J3GP6G 28554344

[B104] Pei S. Cheng H.-M. (2012). The Reduction of Graphene Oxide. Carbon 50 (9), 3210–3228. 10.1016/j.carbon.2011.11.010

[B105] Peng L. Xu Z. Liu Z. Wei Y. Sun H. Li Z. (2015). An Iron-Based green Approach to 1-h Production of Single-Layer Graphene Oxide. Nat. Commun. 6, 5716. 10.1038/ncomms6716 25607686 PMC4354147

[B106] Phaedon A. Christos D. (2012). Graphene: Synthesis and Applications. Mater. Today 15, 83–97. 10.1016/S1369-7021(12)70044-5

[B107] Podolska M. J. Barras A. Alexiou C. Frey B. Gaipl U. Boukherroub R. (2020). Graphene Oxide Nanosheets for Localized Hyperthermia-Physicochemical Characterization, Biocompatibility, and Induction of Tumor Cell Death. Cells 9 (3), 776. 10.3390/cells9030776 PMC7140890 32209981

[B108] Pourhajibagher M. Parker S. Chiniforush N. Bahador A. (2019). Photoexcitation Triggering via Semiconductor Graphene Quantum Dots by Photochemical Doping with Curcumin versus Perio-Pathogens Mixed Biofilms. Photodiagnosis Photodynamic Ther. 28, 125–131. 10.1016/j.pdpdt.2019.08.025 31479805

[B109] Priyadarsini S. Mukherjee S. Mishra M. (2018). Nanoparticles Used in Dentistry: A Review. J. Oral Biol. Craniofac. Res. 8, 58–67. 10.1016/j.jobcr.2017.12.004 29556466 PMC5854556

[B110] Qian W. Qiu J. Liu X. (2019). Minocycline Hydrochloride‐loaded Graphene Oxide Films on Implant Abutments for Peri‐implantitis Treatment in Beagle Dogs. J. Periodontol. 91 (6), 792–799. 10.1002/JPER.19-0285 31782532

[B111] Qian W. Qiu J. Su J. Liu X. (2018). Minocycline Hydrochloride Loaded on Titanium by Graphene Oxide: An Excellent Antibacterial Platform with the Synergistic Effect of Contact-Killing and Release-Killing. Biomater. Sci. 6 (2), 304–313. 10.1039/c7bm00931c 29184938

[B112] Qiu J. Geng H. Wang D. Qian S. Zhu H. Qiao Y. (2017). Layer-Number Dependent Antibacterial and Osteogenic Behaviors of Graphene Oxide Electrophoretic Deposited on Titanium. ACS Appl. Mater. Inter. 9 (14), 12253–12263. 10.1021/acsami.7b00314 28345852

[B113] Rai V. K. Mahata S. Kashyap H. Singh M. Rai A. (2020). Bio-reduction of Graphene Oxide: Catalytic Applications of (Reduced) GO in Organic Synthesis. Cos 17 (3), 164–191. 10.2174/1570179417666200115110403 32538718

[B114] Reina G. González-Domínguez J. M. Criado A. Vázquez E. Bianco A. Prato M. (2017). Promises, Facts and Challenges for Graphene in Biomedical Applications. Chem. Soc. Rev. 46 (15), 4400–4416. 10.1039/c7cs00363c 28722038

[B115] Roos-Jansåker A.-M. Renvert S. Egelberg J. (2003). Treatment of Peri-Implant Infections: A Literature Review. J. Clin. Periodontol. 30 (6), 467–485. 10.1034/j.1600-051X.2003.00296.x 12795785

[B116] Rosa V. Malhotra R. Agarwalla S. V. Morin J. L. P. Luong-Van E. K. Han Y. M. (2021). Graphene Nanocoating: High Quality and Stability upon Several Stressors. J. Dent. Res. 100 (10), 1169–1177. 10.1177/00220345211024526 34253090

[B118] Ruse N. D. Sadoun M. J. (2014). Resin-composite Blocks for Dental CAD/CAM Applications. J. Dent. Res. 93 (12), 1232–1234. 10.1177/0022034514553976 25344335 PMC4462808

[B119] Schünemann F. H. Galárraga-Vinueza M. E. Magini R. Fredel M. Silva F. Souza J. C. M. (2019). Zirconia Surface Modifications for Implant Dentistry. Mater. Sci. Eng. C 98, 1294–1305. 10.1016/j.msec.2019.01.062 PMC6402584 30813009

[B120] Schwarz F. Becker K. Rahn S. Hegewald A. Pfeffer K. Henrich B. (2015). Real-time PCR Analysis of Fungal Organisms and Bacterial Species at Peri-Implantitis Sites. Int. J. Implant Dent 1 (1), 1–7. 10.1186/s40729-015-0010-6 27747631 PMC5005605

[B121] Seonwoo H. Jang K.-J. Lee D. Park S. Lee M. Park S. (2018). Neurogenic Differentiation of Human Dental Pulp Stem Cells on Graphene-Polycaprolactone Hybrid Nanofibers. Nanomaterials 8 (7), 554. 10.3390/nano8070554 PMC6071115 30037100

[B122] Sharan J. Singh S. Lale S. V. Mishra M. Koul V. Kharbanda O. P. (2017). Applications of Nanomaterials in Dental Science: A Review. J. Nanosci Nanotechnol 17 (4), 2235–2255. 10.1166/jnn.2017.13885 29638105

[B123] Shin Y. C. Song S.-J. Song. Jeong S. J. Kim B. Kwon I. K. Hong S. W. (2018). Graphene-Based Nanocomposites as Promising Options for Hard Tissue Regeneration. Adv. Exp. Med. Biol. 1078, 103–117. 10.1007/978-981-13-0950-2_6 30357620

[B125] Slots J. (2017). Periodontitis: Facts, Fallacies and the Future. Periodontol. 2000 75 (1), 7–23. 10.1111/prd.12221 28758294

[B126] Smeets R. Henningsen A. Jung O. Heiland M. Hammächer C. Stein J. M. (2014). Definition, Etiology, Prevention and Treatment of Peri-Implantitis - a Review. Head Face Med. 10, 34. 10.1186/1746-160x-10-34 25185675 PMC4164121

[B127] Song H. S. Kwon O. S. Kim J.-H. Conde J. Artzi N. (2017). 3D Hydrogel Scaffold Doped with 2D Graphene Materials for Biosensors and Bioelectronics. Biosens. Bioelectron. 89 (Pt 1), 187–200. 10.1016/j.bios.2016.03.045 27020065

[B128] Souza J. C. M. Sordi M. B. Kanazawa M. Ravindran S. Henriques B. Silva F. S. (2019). Nano-scale Modification of Titanium Implant Surfaces to Enhance Osseointegration. Acta Biomater. 94, 112–131. 10.1016/j.actbio.2019.05.045 31128320

[B129] Steflik D. E. Corpe R. S. Young T. R. Sisk A. L. Parr G. R. (1999). The Biologic Tissue Responses to Uncoated and Coated Implanted Biomaterials. Adv. Dent Res. 13 (1), 27–33. 10.1177/08959374990130011101 11276743

[B131] Sun L. Yan Z. Duan Y. Zhang J. Liu B. (2018). Improvement of the Mechanical, Tribological and Antibacterial Properties of Glass Ionomer Cements by Fluorinated Graphene. Dental Mater. 34 (6), e115–e127. 10.1016/j.dental.2018.02.006 29567317

[B132] Suo L. Jiang N. Wang Y. Wang P. Chen J. Pei X. (2018). The Enhancement of Osseointegration Using a Graphene Oxide/chitosan/hydroxyapatite Composite Coating on Titanium Fabricated by Electrophoretic Deposition. J. Biomed. Mater. Res. 107 (3), 635–645. 10.1002/jbm.b.34156 29802685

[B133] Tatullo M. Genovese F. Aiello E. Amantea M. Makeeva I. Zavan B. (2019a). Phosphorene Is the New Graphene in Biomedical Applications. Materials 12 (14), 2301. 10.3390/ma12142301 PMC6678593 31323844

[B134] Tatullo M. Zavan B. Genovese F. Codispoti B. Makeeva I. Rengo S. (2019b). Borophene Is a Promising 2D Allotropic Material for Biomedical Devices. Appl. Sci. 9 (17), 3446. 10.3390/app9173446

[B135] Trusek A. Kijak E. (2021). Drug Carriers Based on Graphene Oxide and Hydrogel: Opportunities and Challenges in Infection Control Tested by Amoxicillin Release. Materials 14 (12), 3182. 10.3390/ma14123182 34207735 PMC8228297

[B136] Vera-Sánchez M. Aznar-Cervantes S. Jover E. García-Bernal D. Oñate-Sánchez R. E. Hernández-Romero D. (2016). Silk-fibroin and Graphene Oxide Composites Promote Human Periodontal Ligament Stem Cell Spontaneous Differentiation into Osteo/cementoblast-like Cells. Stem Cell Development 25 (22), 1742–1754. 10.1089/scd.2016.0028 27503546

[B137] Verma S. Singh A. Shukla A. Kaswan J. Arora K. Ramirez-Vick J. (2017). Anti-IL8/AuNPs-rGO/ITO as an Immunosensing Platform for Noninvasive Electrochemical Detection of Oral Cancer. ACS Appl. Mater. Inter. 9 (33), 27462–27474. 10.1021/acsami.7b06839 28766330

[B138] Verma S. Singh S. P. (2019). Non-invasive Oral Cancer Detection from Saliva Using Zinc Oxide-Reduced Graphene Oxide Nanocomposite Based Bioelectrode. MRS Commun. 9 (4), 1227–1234. 10.1557/mrc.2019.138

[B117] Vinicius Rosa Han Nileshkumar Dubey (2016). Graphene Oxide-Based Substrate: Physical and Surface Characterization, Cytocompatibility and Differentiation Potential of Dental Pulp Stem Cells. Dental Mater. 32 (8), 1019–1025. 10.1016/j.dental.2016.05.008 27283997

[B139] Wang G. Qian F. Saltikov C. W. Jiao Y. Li Y. (2011). Microbial Reduction of Graphene Oxide by Shewanella. Nano Res. 4 (6), 563–570. 10.1007/s12274-011-0112-2

[B140] Wang K. Ruan J. Song H. Zhang J. Wo Y. Guo S. (2011). Biocompatibility of Graphene Oxide. Nanoscale Res. Lett. 6 (1), 8. 10.1007/s11671-010-9751-6 27502632 PMC3212228

[B141] Wang Y. Chen Y. Lacey S. D. Xu L. Xie H. Li T. (2018). Reduced Graphene Oxide Film with Record-High Conductivity and Mobility. Mater. Today 21, 186–192. 10.1016/j.mattod.2017.10.008

[B142] Wei C. Liu Z. Jiang F. Zeng B. Huang M. Yu D. (2017). Cellular Behaviours of Bone Marrow-Derived Mesenchymal Stem Cells towards Pristine Graphene Oxide Nanosheets. Cell Prolif 50 (5), e12367. 10.1111/cpr.12367 PMC6529149 28771866

[B143] Wessing B. Lettner S. Zechner W. (2018). Guided Bone Regeneration with Collagen Membranes and Particulate Graft Materials: A Systematic Review and Meta-Analysis. Int. J. Oral Maxillofac. Implants 33 (1), 87–100. 10.11607/jomi.5461 28938035

[B144] Williams G. Seger B. Kamat P. V. (2008). TiO2-Graphene Nanocomposites. UV-Assisted Photocatalytic Reduction of Graphene Oxide. ACS Nano 2 (7), 1487–1491. 10.1021/nn800251f 19206319

[B145] Wu J. Zheng A. Liu Y. Jiao D. Zeng D. Wang X. (2019). Enhanced Bone Regeneration of the Silk Fibroin Electrospun Scaffolds through the Modification of the Graphene Oxide Functionalized by BMP-2 Peptide. Ijn 14, 733–751. 10.2147/IJN.S187664 30705589 PMC6342216

[B146] Wu R. Zhao Q. Lu S. Fu Y. Yu D. Zhao W. (2018). Inhibitory Effect of Reduced Graphene Oxide-Silver Nanocomposite on Progression of Artificial Enamel Caries. J. Appl. Oral Sci. 27, e20180042. 10.1590/1678-7757-2018-0042 30540069 PMC6296285

[B147] Wu X. Ding S.-J. Lin K. Su J. (2017). A Review on the Biocompatibility and Potential Applications of Graphene in Inducing Cell Differentiation and Tissue Regeneration. J. Mater. Chem. B 5 (17), 3084–3102. 10.1039/c6tb03067j 32263706

[B148] Xiao N. Dong X. Song L. Liu D. Tay Y. Wu S. (2011). Enhanced Thermopower of Graphene Films with Oxygen Plasma Treatment. ACS Nano 5 (4), 2749–2755. 10.1021/nn2001849 21417404

[B149] Xie H. Chua M. Islam I. Bentini R. Cao T. Viana-Gomes J. C. (2017). CVD-grown Monolayer Graphene Induces Osteogenic but Not Odontoblastic Differentiation of Dental Pulp Stem Cells. Dental Mater. 33 (1), e13–e21. 10.1016/j.dental.2016.09.030 27692439

[B150] Xie Y. Li H. Zhang C. Gu X. Zheng X. Huang L. (2014). Graphene-reinforced Calcium Silicate Coatings for Load-Bearing Implants. Biomed. Mater. 9 (2), 025009. 10.1088/1748-6041/9/2/025009 24518251

[B151] Yin Z. Sun S. Salim T. Wu S. Huang X. He Q. (2010). Organic Photovoltaic Devices Using Highly Flexible Reduced Graphene Oxide Films as Transparent Electrodes. ACS Nano 4 (9), 5263–5268. 10.1021/nn1015874 20738121

[B152] Yu H. Zhang B. Bulin C. Li R. Xing R. (2016). High-efficient Synthesis of Graphene Oxide Based on Improved Hummers Method. Sci. Rep. 6, 36143. 10.1038/srep36143 27808164 PMC5093679

[B153] Zhang Y. Wan Q. Yang N. (2019). Recent Advances of Porous Graphene: Synthesis, Functionalization, and Electrochemical Applications. Small 15 (48), 1903780. 10.1002/smll.201903780 31663294

[B154] Zhao M. Shan T. Wu Q. Gu L. (2020). The Antibacterial Effect of Graphene Oxide on Streptococcus Mutans. J. Nanosci Nanotechnol 20 (4), 2095–2103. 10.1166/jnn.2020.17319 31492217

[B155] Zhou Q. Yang P. Li X. Liu H. Ge S. (2016). Bioactivity of Periodontal Ligament Stem Cells on Sodium Titanate Coated with Graphene Oxide. Sci. Rep. 6 (1), 19343. 10.1038/srep19343 26763307 PMC4725920

